# GADD45A regulates subcutaneous fat deposition and lipid metabolism by interacting with Stat1

**DOI:** 10.1186/s12915-023-01713-z

**Published:** 2023-10-09

**Authors:** Wenjing You, Shiqi Liu, Jie Li, Yuang Tu, Tizhong Shan

**Affiliations:** 1 College of Animal Sciences, Zhejiang University, Hangzhou, China; 2 The Key Laboratory of Molecular Animal Nutrition, Ministry of Education, Hangzhou, China; 3 Zhejiang Provincial Laboratory of Feed and Animal Nutrition, No. 866 Yuhangtang Road, Hangzhou, 310058 Zhejiang China

**Keywords:** GADD45A, Obesity, Subcutaneous fat, Lipid metabolism, Adipogenesis, Stat1

## Abstract

**Background:**

Obesity, characterized by excessive white adipose tissue expansion, is associated with several metabolic complications. Identifying new adipogenesis regulators may lead to effective therapies for obesity-induced metabolic disorders.

**Results:**

Here, we identified the growth arrest and DNA damage-inducible A (GADD45A), a stress-inducible histone-folding protein, as a novel regulator of subcutaneous adipose metabolism. We found that GADD45A expression was positively correlated with subcutaneous fat deposition and obesity in humans and fatty animals. In vitro, the gain or loss function of GADD45A promoted or inhibited subcutaneous adipogenic differentiation and lipid accumulation, respectively. Using a *Gadd45a*^*-/-*^ mouse model, we showed that compared to wild-type (WT) mice, knockout (KO) mice exhibited subcutaneous fat browning and resistance to high-fat diet (HFD)-induced obesity. GADD45A deletion also upregulated the expression of mitochondria-related genes. Importantly, we further revealed that the interaction of GADD45A with Stat1 prevented phosphorylation of Stat1, resulting in the impaired expression of Lkb1, thereby regulating subcutaneous adipogenesis and lipid metabolism.

**Conclusions:**

Overall, our results reveal the critical regulatory roles of GADD45A in subcutaneous fat deposition and lipid metabolism. We demonstrate that GADD45A deficiency induces the inguinal white adipose tissue (iWAT) browning and protects mice against HFD-induced obesity. Our findings provide new potential targets for combating obesity-related metabolic diseases and improving human health.

**Supplementary Information:**

The online version contains supplementary material available at 10.1186/s12915-023-01713-z.

## Background

Obesity is a complex pathophysiology closely associated with chronic metabolic diseases such as type 2 diabetes, insulin resistance, cardiovascular disease, and non-alcoholic fatty liver disease and has become a major health burden with a rapid global epidemic [1–4]. In farm animals, excessive lipid accumulation can affect carcasses’ leanness and reduces their economic value. Therefore, there is an urgent need to develop effective therapeutic strategies to reduce obesity. Adipose tissue is composed of mature adipocytes and preadipocytes and plays a vital role in regulating whole-body glucose homeostasis and energy metabolism. Three types of adipocytes have been identified in mammals, including white, brown, and beige adipocytes [5]. White adipocytes are the primary cell type in white adipose tissue (WAT), store triglyceride as energy, and are closely related to obesity [6]. Brown adipocytes are the major cells of brown adipose tissue (BAT), contain abundant mitochondria, and highly express uncoupling protein 1 (UCP1) [7]. Beige adipocytes identified in subcutaneous adipose tissue (SAT) also have abundant mitochondria and high levels of UCP1 expression. Both brown and beige fat cells can burn lipids into heat, improving energy expenditure and combating obesity [7]. Thus, understanding the molecular regulation of adipose tissue may provide a potential strategy for alleviating obesity and other metabolic complications.

The growth arrest and DNA damage 45A (GADD45A) is a p53-regulated histone folding protein induced by various cellular stresses [8]. Previous evidence has revealed the crucial role of GADD45A in DNA repair, cell cycle, apoptosis, DNA demethylation, and energy metabolism [9, 10]. *Gadd45a*^*-/-*^ mice display increased genomic instability and a pronounced metabolic phenotype [9, 11]. The GADD45A/ING1 double-knockout (KO) mice exhibit partial premature aging, lipodystrophy, and several metabolic abnormalities [12]. GADD45A affects mitochondrial function and myofiber size in skeletal muscle [13, 14] and regulates osteogenic and adipogenic differentiation of adipose-derived mesenchymal stem cells [15, 16]. Our previous study showed that GADD45A affects the thermogenesis of brown adipocytes [9] and promotes intramuscular fat infiltration [17]. These findings suggest that GADD45A might be an important candidate gene for animal growth and glucose and lipid metabolism. However, the specific mechanism and interacting proteins of GADD45A in the adipogenic differentiation of subcutaneous preadipocytes remain to be elucidated.

Here, we identified GADD45A as a novel regulator of WAT plasticity and lipid metabolism. We found that GADD45A expression is positively associated with subcutaneous fat deposition and obesity in humans and animals. In vitro, the gain or loss function of GADD45A promotes or inhibits subcutaneous preadipocyte adipogenesis. Using the *Gadd45a*^*-/-*^ mice model, we showed that compared with wild-type (WT) littermates, the KO mice exhibit subcutaneous fat browning and attenuate high-fat diet (HFD)-induced obesity. At the molecular level, GADD45A transcriptionally regulates liver kinase b1 (Lkb1) by interacting with signal transducer and activator of transcription 1 (Stat1) to regulate subcutaneous adipogenesis and lipid metabolism. Our findings provide new potential targets for treating obesity-related metabolic diseases and improving human and animal health.

## Results

### GADD45A expression is positively associated with subcutaneous fat deposits in fatty animals

**Fig. 1:**
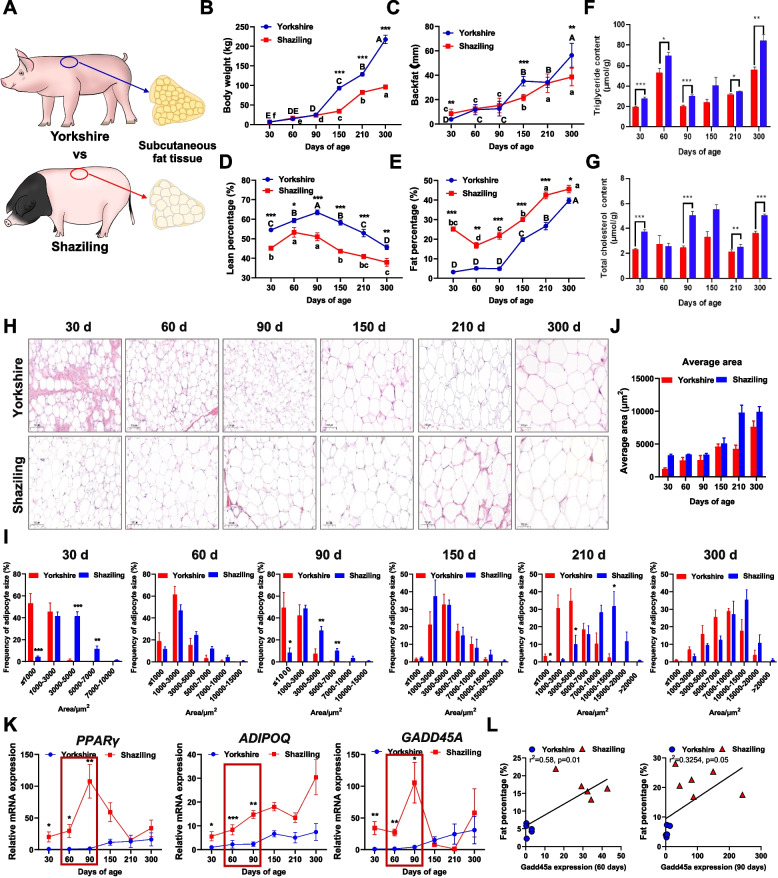
GADD45A expression is positively associated with subcutaneous fat deposits and lipid metabolism in Shaziling and Yorkshire pigs. **A** Schematic diagram of subcutaneous fat in Shaziling and Yorkshire pigs. **B**–**E** Carcass traits of Body weight (**B**), backfat (**C**), lean percentage (**D**), and fat percentage (**E**) between the two breeds. ^a to e^ Different letters denote differences among Shaziling pigs (*P* < 0.05). ^A to E^ Different letters represents differences among Yorkshire pigs (*P* < 0.01) (*n* = 6). **F**, **G** Triglyceride (TG) and total cholesterol (TC) are shown (*n* = 6). **H** Comparison of the development of subcutaneous adipocytes in Shaziling and Yorkshire pigs by Hematoxylin and eosin (H&E) staining. **I**, **J** The cell area was quantified using Image J software (*n* = 6). **K** Relative mRNA expression of *PPARγ*, *ADIPOQ*, and *GADD45A* (*n* = 6). **L** Correlation analyses of *GADD45A* and fat percentage (60 days and 90 days) in the two breeds (*n* = 6). Error bars represent SEM, *P < 0.05, **P < 0.01, ***P < 0.001, two-tailed Student’s *t*-test. Scale bars: 100 μm

We first compared fat deposition in fatty and lean animals to identify the candidate genes that specifically regulate subcutaneous fat deposition. Pigs are non-primate mammals that are very similar to humans in terms of anatomy, genetics, physiology, and lipid metabolism and can serve as excellent model organisms for studying glycolipid metabolism [18, 19]. In this study, we re-analyzed and revealed the carcass traits of Shaziling (fatty) and Yorkshire (lean) pigs at different growth stages [20] (Fig. 1A–E). Our in vivo studies showed that body weight and backfat thickness increased with age (*P* < 0.05, Fig. 1B, C). Shaziling pigs had significantly lower body weight and backfat thickness than Yorkshire pigs at 150 and 300 days of age (*P* < 0.05, Fig. 1B, C). With the increase of age, the lean percentage of Shaziling pigs and Yorkshire pigs was first increased and then decreased (*P* < 0.05, Fig. 1D), while the fat rate of Shaziling pigs showed an opposite trend (*P* < 0.05, Fig. 1E). Throughout the experiment, Shaziling pigs had greater fat deposition capacity and higher triglycerides (TGs) and total cholesterol (TC) than Yorkshire pigs (Fig. 1F, G). To elucidate the developmental pattern of subcutaneous adipocytes in the two breeds, we performed hematoxylin and eosin (H&E) staining and counted the size of the subcutaneous adipocyte area. The subcutaneous adipocyte area increased with age in both breeds, and the average adipocyte area was significantly larger in Shaziling pigs than in Yorkshire pigs (Fig. 1H–J). Moreover, the mRNA expression levels of mature adipocyte marker genes (*FABP4*, *ADIPOQ*, and *LEP*), adipogenic differentiation-related genes (*CEBPα* and *PPARγ*), fatty acid synthesis gene (*FASN*), and *GADD45A* were significantly upregulated in 30, 60, and 90 days of Shaziling pigs (Fig. 1K; Additional file 1: Fig. S1A-C). Notably, GADD45A expression was positively correlated with fat percentage (Fig. 1L). These results suggest that GADD45A expression is positively associated with subcutaneous fat deposition in fatty animals.

### GADD45A is highly expressed in both human and mouse subcutaneous WAT

**Fig. 2:**
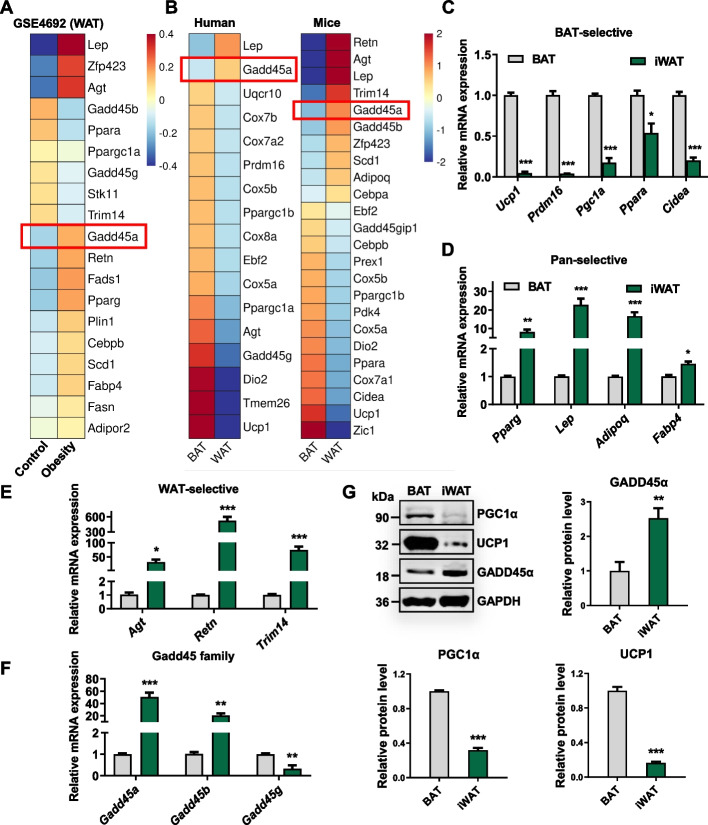
GADD45A is highly expressed in human and mouse subcutaneous WAT. **A** Heatmap of differentially expressed genes (DEGs) in WAT in mice obesity model (GSE4692, PMID:16733553). **B** Heatmap of DEGs in BAT and WAT in humans and mice (PMID:30332656). **C**–**F** qPCR analysis of BAT-selective (**C**), pan-selective (**D**), WAT-selective (**E**), and Gadd45 family (**F**) in BAT and iWAT of WT mice (*n* = 4). **G** Western blotting for PGC1α, UCP1, and GADD45A in BAT and iWAT of WT mice. The quantification of protein levels was performed densitometrically and normalized to GAPDH levels (*n* = 4). Error bars represent SEM, *P < 0.05, **P < 0.01, ***P < 0.001, two-tailed Student’s *t*-test

To consider the possible role of GADD45A in lipid metabolism, we reanalyzed publicly available microarray data (GSE4692) from human [21] and mouse [21, 22] adipose tissue. Among these differentially expressed genes (DEGs), we found that GADD45A is highly expressed in subcutaneous WAT in mice and humans and is associated with obesity (Fig. 2A, B). BAT-specific genes (*Ucp1*, *Pgc1a*, *Prdm16*, *Cidea*) and mitochondria-related genes (*Cox5a*, *Cox7b*, *Cox8a*, *Uqcr10*) were expressed at lower levels in inguinal WAT (iWAT) (Fig. 2C), whereas Pan-selective (*Pparg*, *Lep*, *Adipoq*) and WAT-selective genes (*Agt*, *Retn*, *Trim14*) were expressed at higher levels (Fig. 2D, E). We showed that the mRNA expression level of *Gadd45a* was significantly higher in iWAT than in BAT, which was elevated approximately 50-fold (Fig. 2F), and the protein level of GADD45A was also markedly higher in iWAT than in BAT (Fig. 2G). These results are consistent with transcriptome analysis and suggest GADD45A is highly expressed in human and mouse subcutaneous WAT.

### GADD45A affects subcutaneous adipogenic differentiation

**Fig. 3:**
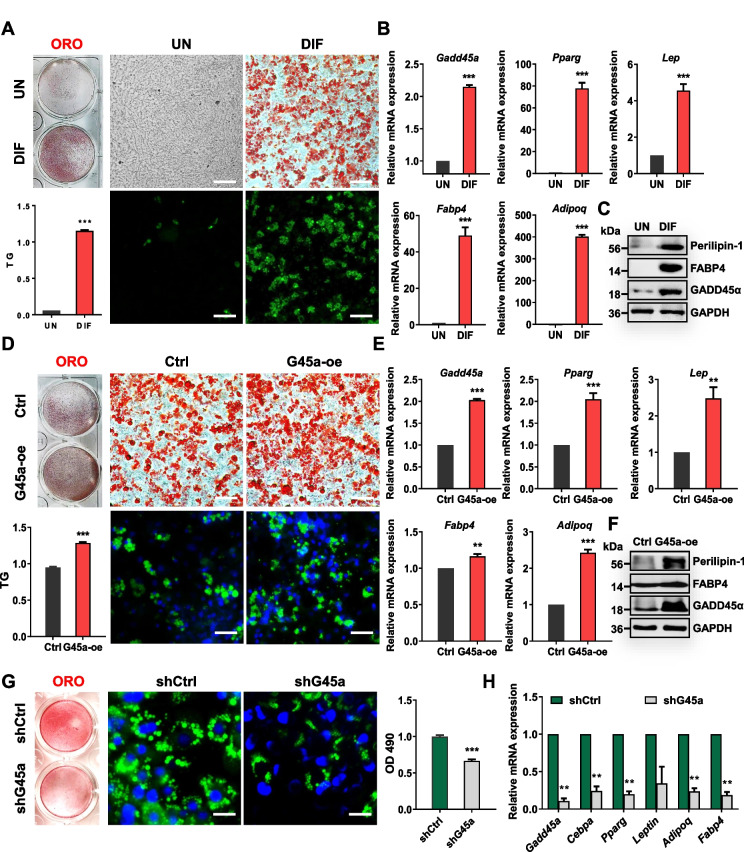
GADD45A affects adipogenesis in white preadipocytes. **A** ORO and BODIPY staining of total lipids in undifferentiated and differentiated mice subcutaneous preadipocytes. TG was measured (*n* = 3). **B**, **C** The expression of adipogenic genes and GADD45A was assessed by qPCR (**B**) (*n* = 3) and Western blotting (**C**). **D** Mice subcutaneous adipocytes were infected with control adenovirus (Ctrl) and adenovirus-expressing GADD45A (G45a-oe) and differentiated for 6 days. ORO and BODIPY staining of control and G45a-oe cells after induction of differentiation. Nuclei were stained with DAPI. TG was measured (*n* = 3). **E**, **F** qPCR and western blotting for the expression of GADD45A and adipogenic-related genes in control and G45a-oe cells (*n* = 3). **G** ORO and BODIPY staining in differentiated preadipocytes infected with mouse control shRNA (shCtrl) and GADD45A shRNA (shG45a). Nuclei were stained with DAPI (blue). OD490 was measured (*n* = 6). **H** The mRNA levels of related genes with GADD45A KD after differentiation (*n* = 4). Error bars represent SEM, *P < 0.05, **P < 0.01, ***P < 0.001, two-tailed Student’s *t*-test. Scale bars: 100 μm and 50 μm

To determine the regulatory role of GADD45A in subcutaneous fat deposition, we isolated both porcine and mouse subcutaneous preadipocytes and subjected them to lipogenic induction. Oil red O (ORO) and BODIPY staining as well as triglyceride measurements showed that fat cells undergo adipogenic differentiation with significant lipid accumulation (Fig. 3A; Additional file 2: Fig. S2A). The mRNA levels of adipogenesis-related genes (*PPARγ*, *LEP*, *FABP4*, and *ADIPOQ*) were significantly upregulated (Fig. 3B; Additional file 2: Fig. S2B). The mRNA and protein levels of GADD45A were also dramatically increased, confirming that GADD45A was expressed in mature adipocytes (Fig. 3B, C; Additional file 2: Fig. S2B, C). We performed gain-of-function and loss-of-function experiments. GADD45A overexpressing (G45a-oe) adipocytes accumulated higher levels of TGs and upregulated the mRNA levels of *PPARγ*, *LEP*, *FABP4*, and *ADIPOQ*, as well as protein levels of Perilipin-1 (Fig. 3D–F; Additional file 2: Fig. S2D-F). In contrast, GADD45A knockdown (shG45a) potently reduced lipid accumulation (Fig. 3G, H; Additional file 2: Fig. S2G-I). Taken together, we concluded that GADD45A promotes subcutaneous adipocyte differentiation and lipid accumulation in murine and porcine adipocytes.

### GADD45A deletion promotes WAT browning and protects against HFD-induced obesity

**Fig. 4:**
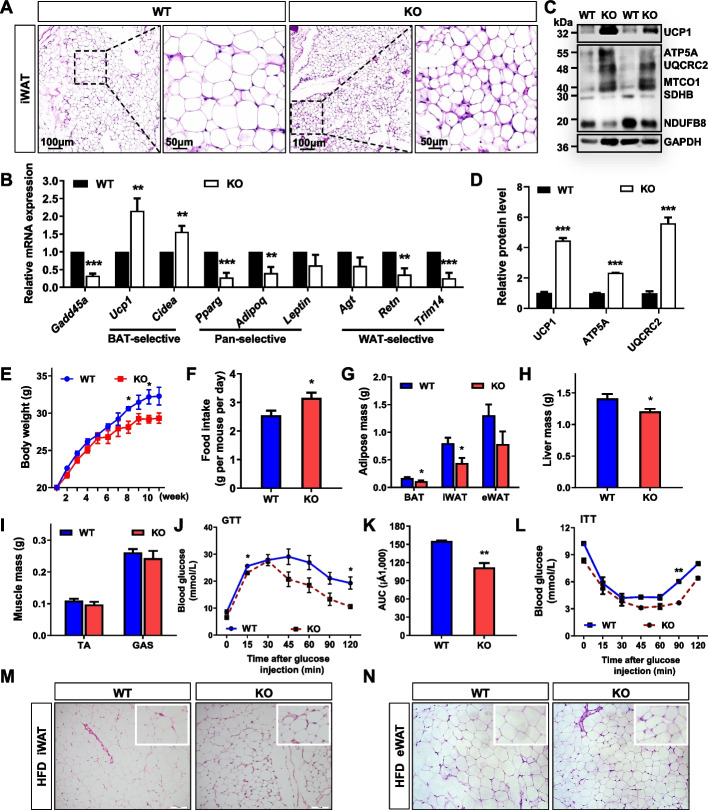
Ablation of GADD45A promotes browning of subcutaneous WAT and protects mice against high-fat diet (HFD)-induced obesity. **A** H&E staining of iWAT sections from WT and KO mice at eight weeks. Scale bar, 100 μm and 50 μm, respectively. **B** Relative mRNA expression of pan-adipocyte, BAT- and WAT-selective genes in iWAT from WT and KO mice (*n* = 6). **C** The protein levels of UCP1, ETC (electron transport chain) complexes (ATP5A, ATP synthase, H^+^ transporting, mitochondrial F1 complex, alpha 1; UQCRC2, ubiquinol-cytochrome c reductase core protein II; MTCO1, cytochrome c oxidase I; SDHB, succinate dehydrogenase complex iron sulfur subunit B; NDUFB8, ubiquinone oxidoreductase subunit B8) in iWAT of WT and KO mice. **D** The quantification of protein levels is based on **C** (*n* = 6). **E**–**I** Body weight (**E**), food intake (**F**), adipose mass (**G**), liver mass (**H**), and muscle mass (**I**) in WT and KO mice after HFD for 10 weeks (*n* = 6). **J**, **K** Blood glucose concentrations (**J**) and calculated area under the curve (AUC) (**K**) during glucose tolerance tests (GTT) performed in WT and KO male mice after HFD (*n* = 6). **L** Blood glucose concentrations during insulin tolerance tests (ITT) were performed in WT and KO male mice (*n* = 6). **M**, **N** H&E staining of iWAT and eWAT sections from WT and KO mice after HFD. Error bars represent SEM, *P < 0.05, **P < 0.01, ***P < 0.001, two-tailed Student’s *t*-test. Scale bars: 100 μm and 50 μm

To further investigate the function of GADD45A in SAT, we used the *Gadd45a*^*-/-*^ mouse model. H&E staining showed that KO mice had smaller lipid droplets in iWAT with significantly smaller cell volume and some multi-chambered adipocytes with a tendency of subcutaneous adipose browning. In comparison, WT mice had mature subcutaneous adipocytes with larger cell morphology and lipid droplets (Fig. 4A). The qPCR results showed that GADD45A KO significantly promoted the mRNA expression of BAT-selective genes (*Ucp1* and *Cidea*) and downregulated the expression of Pan-selective (*Pparg* and *Adipoq*) and WAT-selective (*Retn* and *Trim14*) genes in iWAT (Fig. 4B). The protein levels of UCP1 and mitochondrial protein complexes (UQCRC2 (III), MTCO1 (IV), and ATP5A (V)) were found to be significantly upregulated in iWAT of KO mice (Fig. 4C, D). The above results indicated that GADD45A ablation might induce iWAT browning, inhibit subcutaneous adipocyte differentiation, and affect mitochondrial gene expression.

To examine the long-term effects of GADD45A deletion on energy metabolism, we administered an HFD to WT and KO mice. After HFD feeding for more than 10 weeks, KO mice had lower body weight, fat (BAT and iWAT), and liver masses than WT mice but higher food intake (Fig. 4E–H). Other tissues, including the tibialis anterior muscle (TA) and gastrocnemius muscle (GAS), were unchanged (Fig. 4I). Notably, *Gadd45a*^*-/-*^ mice maintained better glucose tolerance and lower insulin resistance after HFD feeding (Fig. 4J–L). We performed H&E staining and calculated the size of adipocyte area in iWAT and eWAT (Fig. 4M, N). The KO mice had a significantly higher number of smaller adipocyte areas (< 2000 μm^2^) compared to WT mice, while their larger adipocyte areas (> 4000 μm^2^) were lower (Additional file 3: Fig. S3A, C). Additionally, the average adipocyte size was also markedly reduced in KO mice (Additional file 3: Fig. S3B, D). Thus, our results confirm that GADD45A deficiency can protect against HFD-induced obesity.

### The association of GADD45A and Stat1 suppresses the LKB1 expression

**Fig. 5:**
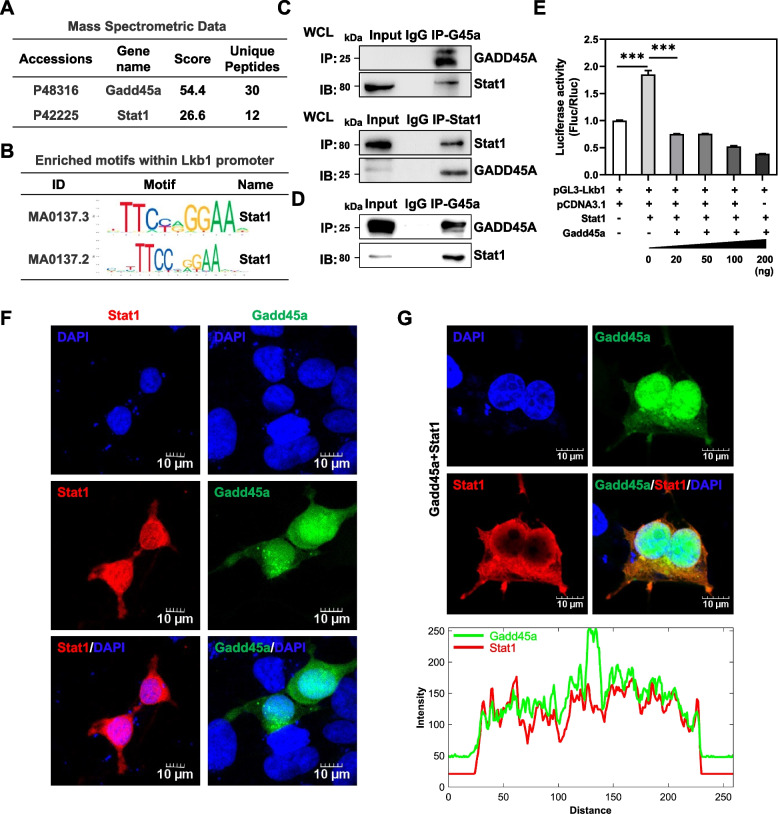
GADD45A suppressed LKB1 expression by interacting with Stat1. **A** Analysis of the GADD45A-associated proteins through mass spectrometry (MS) data. **B** Stat1 binding site prediction in the LKB1 promoter region using JASPAR. **C** Co-IP analysis of the binding between exogenous GADD45A and exogenous Stat1 in HEK293T cells co-transfected with Flag-GADD45A plasmid and HA-Stat1 plasmids. **D** Endogenously expressed GADD45A interacts with Stat1. Cell lysates from differentiated white adipocytes were IP with GADD45A or Stat1 antibodies and blotted with these antibodies. **E** Promoter activity of Lkb1 upon overexpression of GADD45A and/or Stat1 was detected by luciferase reporter assay. HEK293T cells seeded overnight in 24-well plates were co-transfected with pGL3-Lkb1, pCDNA3.1, Stat1, and varying amounts of GADD45A plasmids. The luciferase activities were examined after 24 h (*n* = 3). **F** Confocal immunofluorescence imaging of the subcellular localization of Stat1 (Red) and GADD45A (Green) in HEK293T cells 24 h after transfection with vectors expressing both proteins. Nuclei were stained with DAPI (blue). **G** Examination of the interaction of GADD45A with Stat1 protein by colocalization analysis. HEK293T cells were co-transfected with Stat1 and GADD45A plasmids, and 24 h later, cells were fixed with 4% (v/v) PFA, stained with DAPI and anti-Stat1 antibody, and analyzed by confocal microscopy. The intensity profiles of the indicated proteins along the plotted lines, as analyzed by ImageJ line scan analysis. Error bars represent SEM, *P < 0.05, **P < 0.01, ***P < 0.001, two-tailed Student’s *t*-test. Scale bars: 10 μm

To determine the mechanism underlying GADD45A’s function during subcutaneous adipogenesis, we further analyzed the potential GADD45A-associated proteins from mass spectrometry (MS) data reported by a previous study [23]. The MS data showed that Stat1, a transcriptional regulation factor, strongly interacts with GADD45A (Fig. 5A). We analyzed the binding sites of Stat1 through the online database JASPAR (https://jaspar.genereg.net/) and found that the promoter of liver kinase B1 (LKB1) contains the above binding site (Fig. 5B). LKB1, also named as serine/threonine kinase 11 (STK11), is a primary upstream kinase of adenosine monophosphate-activated protein kinase (AMPK), is important for cell metabolism and energy homeostasis [24, 25]. Our previous studies showed that adipocyte-specific deletion of Lkb1 promotes iWAT SVF cell differentiation, lipid accumulation, and thermogenesis [5]. Conversely, Lkb1 overexpression suppressed adipogenesis in iWAT SVF cells and intramuscular adipocytes [5, 26]. We observed elevated Lkb1 expression in the iWAT of Gadd45a^-/-^ mice compared to that of WT mice (Additional file 4: Fig. S4A, B). These data suggest a possible link between the GADD45A-Stat1 interaction and Lkb1, and we hypothesize that the binding of GADD45A and Stat1 may affect subcutaneous adipogenesis by inhibiting LKB1 expression. We next performed co-immunoprecipitation (Co-IP) experiments to examine whether GADD45A interacts with Stat1. After GADD45A overexpression in HEK293T, we found that Stat1 can be pulled down by GADD45A and vice versa (Fig. 5C). Likewise, endogenous interactions between GADD45A and Stat1 were also found in differentiated 3T3-L1 cells (Fig. 5D). In addition, luciferase reporter assays were performed to examine the promoter activity of LKB1 upon GADD45A and/or Stat1 overexpression. Notably, the promoter activity of LKB1 was significantly enhanced in Stat1 overexpressing cells, while co-transfection of GADD45A and Stat1 markedly attenuated LKB1 promoter activity in a concentration-dependent manner (Fig. 5E). Next, we examined the subcellular location of GADD45A and Stat1 by transiently transfecting GADD45A and Stat1 overexpressing plasmids into HEK293 cells. Immunofluorescence microscopy verified that both GADD45A and Stat1 distributed in the nucleus and cytoplasm when GADD45A or Stat1 were transfected, respectively (Fig. 5F). Interestingly, we found that when these two vectors were co-transfected into HEK293 cells, Stat1 expression was mainly in the cytoplasm and co-localized with GADD45A (Fig. 5G). The intensity profiles of GADD45A and Stat1 along the plotted lines were consistent with the observations by confocal microscopy (Fig. 5G). These results indicated that GADD45A may prevent translocation of Stat1 into the nucleus by interacting with Stat1 in the cytoplasm.

**Fig. 6:**
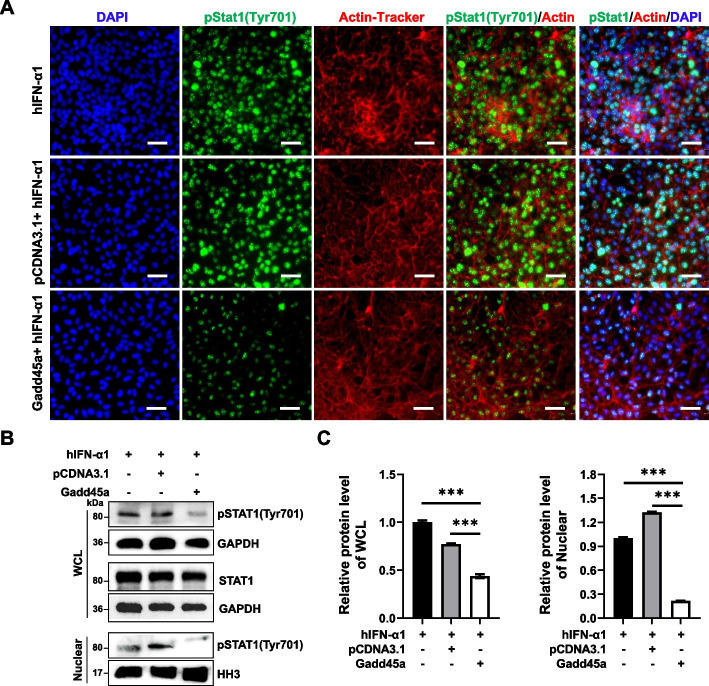
GADD45A prevented the phosphorylation of Stat1. **A** Immunofluorescence staining of phosphorylation of Stat1 in HEK293T cells. Cells were transfected with pCDNA3.1 or GADD45A plasmid. 24 h later, cells were treated for 30 min with hIFN-α1. Then, cells were stained with primary anti-pStat1 (Tyr701) and secondary species-specific FITC-conjugated antibodies, followed by Actin-Tracker and DAPI. **B** Western blotting analysis of whole cell lysate (WCL) and nuclear fractions in HEK293T cells with hIFN-α1 treatment. **C** Relative gray values for the phosphorylation of Stat1 (Tyr701) in **B** (*n* = 3). Error bars represent SEM, *P < 0.05, **P < 0.01, ***P < 0.001, two-tailed Student’s *t*-test. Scale bars: 100 μm

To test the above hypothesis, we examined the effect of GADD45A on Stat1 phosphorylation by immunofluorescence staining and Western blotting. To this end, HEK293 cells were treated with hIFNα-1, which has been reported to promote the phosphorylation of Stat1 [27]. After treatment with hIFNα-1 for 30 min, Stat1 in most cells was successfully phosphorylated as determined by the nuclear localization of Stat1, while phosphorylation of Stat1 was impaired after GADD45A overexpression (Fig. 6A). The results were also confirmed by Co-IP experiment (Fig. 6B, C). These results indicated that the interaction of GADD45A with Stat1 prevented phosphorylation of Stat1, resulting in the impaired expression of LKB1.

## Discussion

**Fig. 7:**
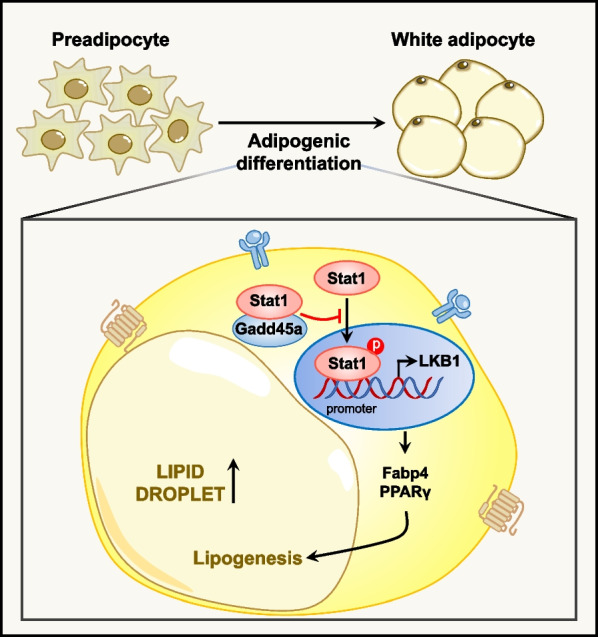
Schematic diagram showing that GADD45A regulates subcutaneous fat deposition by interacting with Stat1

In this study, we found that GADD45A is highly expressed in human and animal SAT and is associated with metabolic diseases such as obesity. We showed that deletion of GADD45A significantly increases Ucp1 expression and induces iWAT browning, protects mice from HFD-induced obesity, and improves glucose tolerance and insulin sensitivity. We elucidated that GADD45A affects subcutaneous adipocyte differentiation and metabolism through the Stat1/Lkb1 pathway and provided biochemical evidence that GADD45A interacts with Stat1 and that GADD45A overexpression inhibits Stat1 phosphorylation and thus Lkb1 transcriptional activity (Fig. 7). Our results reveal a critical role for GADD45A in regulating subcutaneous WAT development and energy metabolism.

Using unbiased transcriptome data analysis, we found that GADD45A is highly expressed in human and mouse SATs and is associated with obesity. By analyzing the transcriptome of human white and brown adipose tissue biopsies, we found that GADD45A is expressed at lower levels in supraclavicular BAT (GSE113764, Additional file 5: Fig. S5A). Additionally, analysis of human gene expression profiles of non-thermic and thermogenic adipose tissue demonstrated that supraclavicular adipose tissue (SClav) exhibits greater expression of thermogenic genes and lower expression of GADD45A (GSE150119, Additional file 5: Fig. S5B). GADD45A expression decreases in forskolin-treated mature human adipocytes (GSE65190, Additional file 5: Fig. S5C). It is hypothesized that GADD45A expression is downregulated in thermogenic adipocytes.

By comparing the carcass indexes between fatty (Shaziling) and lean (Yorkshire) breeds, we found that body weights of Yorkshire and Shaziling pigs differed significantly from day 150. As a fatty breed, Shaziling pigs had a higher fat percentage than Yorkshire pigs at all six stages, as well as a thicker backfat at 30, 150, and 300 days. However, the leanness of Shaziling pigs was lower than that of Yorkshire pigs at the whole stage, which is consistent with previous comparisons of local and lean pigs [28, 29]. Comparison of subcutaneous adipocyte size revealed that adipocyte area in Yorkshire and Shaziling pigs had a normal distribution at all stages. The adipocyte size of Shaziling pigs exhibited increased hypertrophy at 150 days compared to 90 days. By analyzing the slopes of increase in adiposity and backfat thickness over time, it was concluded that the critical period for subcutaneous fat deposition in Shaziling pigs was 90–150 days, whereas in Yorkshire pigs the critical period was slightly later, 150–210 days. Shaziling pigs had significantly higher TG and TC content and increased lipid accumulation in subcutaneous fat. Importantly, we revealed that GADD45A gene expression is closely and positively associated with subcutaneous fat deposition in fatty pigs and may serve as a key candidate gene for regulating subcutaneous adipocyte development in pigs. The reported studies so far are only about the cloning of the porcine GADD45A gene sequence, and nothing has been found about the gene structure and regulation of fat cell differentiation.

Lipid droplets (LDs) are storage organelles at the center of lipid and energy balance [30]. The size of LDs determines their capacity for lipid storage. Our study found that mouse and porcine adipocyte progenitors exhibited visible lipid droplet morphology, higher TG content, and significantly increased adipogenic marker gene expression after differentiation. Furthermore, GADD45A overexpression significantly promoted adipogenesis in subcutaneous adipose precursor cells and upregulated the expression of lipogenic marker genes PPARγ and FABP4, while GADD45A interference significantly inhibited lipid accumulation in subcutaneous adipocytes.

We used *Gadd45a*^*-/-*^ mice to investigate the function of GADD45A in SAT. We previously found no significant differences in body weight and morphology in GADD45A KO mice compared to WT mice, but KO mice had significantly reduced iWAT weight [9]. We found that GADD45A KO upregulated the expression of brown-related genes (*Ucp1* and *Cidea*) in iWAT, which affected mitochondrial protein expression and thus induced WAT browning. It has been shown that brown fat and white fat browning have higher oxidative metabolism and can increase thermogenesis through UCP1 uncoupling [7]. Other factors that cause WAT browning include β-aminobutyric acid, γ-aminobutyric acid, PPARɣ agonists, JAK inhibitors, and IRISIN [31, 32]. The Musclin and TFAM factors can also trigger WAT browning [31]. Browning produces beige adipocytes and brown adipocytes with similar properties, rich in mitochondria, and both increase heat production in the form of UCP1 uncoupling. Browning-induced beige adipocyte formation has two sources: (1) White adipocytes are converted to beige adipocytes by cold stimulation; (2) Beige adipocyte progenitors are present in white adipose tissue and can differentiate into mature adipocytes. Given that *Gadd45a*^*-/-*^ mice are systemic KO mice, the formation of beige adipocytes in KO mice may result from white adipocyte conversion of adipose tissue, but a role for adipose tissue paracrine action cannot be excluded. Further studies are needed to investigate the specific origin of GADD45A KO-generated beige adipocytes.

We found that GADD45A KO mice were resistant to HFD-induced obesity and increased the level of energy metabolism in the organism. To investigate the impact of GADD45A KO on systemic metabolism, we previously assessed food intake, energy expenditure, and physical activity in mice using metabolic cages. We observed a marked rise in food intake and VO2 and VCO2 rates as well as an increase in overall activity in *Gadd45a*^*-/-*^ mice compared to WT mice under chow diet conditions [9]. In the present study, we observed that *Gadd45a*^*-/-*^ mice exhibited reduced body weight, improved insulin sensitivity, and increased food intake when fed HFD. These favorable effects may be attributed to the upregulation of BAT-selective genes expression and enhanced mitochondrial biogenesis in the adipose tissue of *Gadd45a*^*-/-*^ mice. Energy expenditure is determined by body size, body composition, food intake, and physical activity. Among these factors, activity-induced energy expenditure is the most variable component of total energy expenditure [33]. In addition, changes in the central nervous system may lead to increased food intake and physical activity, which play a critical role in maintaining adequate energy balance [34]. Sympathetic activation also increases lipolysis and thermogenesis in BAT and other central and peripheral pathways, resulting in higher energy expenditure [35].

We further explored the molecular mechanism. Studies have shown that STATs dimerize, translocate to the nucleus, and bind to specific recognition sequences in the promoter regions of specific target genes, thereby activating or repressing transcription [36]. Stat1 in the cytoplasm can be phosphorylated under the stimulation of extracellular signals and then enter the nucleus to promote the transcription of target genes [37]. In the current study, we showed that GADD45A interacted with Stat1 in the cytoplasm and prevented phosphorylation of Stat1, suppressing the expression of LKB1. LKB1 is a serine/threonine kinase involved in cell metabolism, proliferation, polarity and migration, muscle regeneration, and adipocyte differentiation [38–40]. Our results elucidate the molecular mechanism by which GADD45A regulates Lkb1 expression by interacting with Stat1 at the transcriptional level. Previous studies mainly described GADD45A as a nuclear factor involved in DNA demethylation [41–43]. However, the function of GADD45A in the cytoplasm is still limited. Our results reveal an important role of cytoplasmic GADD45A in regulating adipocyte differentiation, greatly enriching current knowledge about this gene.

## Conclusions

In conclusion, our results reveal the critical regulatory roles of GADD45A in subcutaneous fat deposition and lipid metabolism. We demonstrate that GADD45A deficiency induces iWAT browning and protects mice against HFD-induced obesity. Our findings provide novel insights into the regulatory mechanism of GADD45A and combating obesity-related metabolic diseases.

## Methods

### Animal and sample collection

The experiments involving pigs were approved by the Animal Care Committee of the Institute of Subtropical Agriculture, the Chinese Academy of Sciences, under ethic approval number ISA-2020-023. A total of 72 healthy male pigs (36 Shaziling pigs and 36 Yorkshire pigs) were selected and kept on the same diets and under the same environmental conditions. After overnight fasting, samples were slaughtered at 30, 60, 90, 150, 210, and 300 days of age. Six pigs were available for each age group. Live weight, backfat thickness, leanness, and fat percentage were measured [20], and subcutaneous fat tissue was collected for subsequent analysis.

All procedures involving mice were approved by the Zhejiang University Animal Care and Use Committee. *Gadd45a*^*-/-*^ mice [11] were directly contributed by Professor Albert J. Fornace Jr. (Gene Response Section, DBS, National Cancer Institute, USA) and were maintained on a C57BL/6 background. All mice used in this study, the *Gadd45a*^*-/-*^ mice and their WT littermate controls, were produced from intercrossing *Gadd45a*^*+/-*^ mice obtained from Hangzhou Normal University. Mice were in a C57BL/6J background and housed in the animal facility with free access to water and standard rodent chow food or HFD (D12492, Research Diets, Inc.). The mice were between 8 and 10 weeks in the experiments. PCR genotyping was carried out as described by the supplier [44]; mouse genotyping primer sequences were shown in Additional file 6: Table S1. Food intakes were measured by weighing total individual food consumption once per week.

### Extraction and quantification of TG and TC

Total triglyceride (TG) and total cholesterol (TC) kits were purchased from Nanjing Jiancheng Bioengineering Institute (Nanjing, China). TG and TC in extracted subcutaneous fat samples were measured according to the manufacturer’s protocols. TG and TC levels were normalized to the fat weight.

### Blood glucose measurements

For glucose tolerance tests (GTT), mice were injected i.p. with 100 mg ml^−1^ d-glucose (2 g kg^−1^ body weight) after overnight fasting. Blood glucose was measured at 0, 15, 30, 45, 60, 90, and 120 min via the tail vein by a glucometer (Accu-Check Active, Roche). For insulin tolerance tests (ITT), mice were fasted for 4 h before i.p. administration of human insulin (Santa Cruz) (0.75 U per kg body weight), and tail blood glucose concentrations were monitored.

### H&E and immunostaining

Hematoxylin and eosin (H&E) staining was performed as described previously [5]. For immunostaining, cells were fixed with 4% paraformaldehyde (PFA) and incubated with a blocking buffer containing 5% goat serum, 2% BSA, 0.2% Triton X-100, and 0.1% sodium azide in PBS for 1 h. Then, the samples were incubated with Stat1 (Cell Signaling Technology, D4Y6Z, 1:200), GADD45A (Santa Cruz, sc-6850, 1:200), and Phospho-Stat1 (Tyr701) (Cell Signaling Technology, 58D6, 1:100) primary antibodies at 4 °C overnight. After washing with PBS three times, cells were incubated with secondary antibodies and DAPI for 45 min at room temperature. Fluorescent images were obtained with a confocal microscope (LSM 510 META, Zeiss).

### Adenovirus infection

Control-adenovirus (Ctrl), mouse or porcine Gadd45a-overexpressing adenovirus (G45a-oe), mouse or porcine lentiviral shRNA targeting Gadd45a (shG45a), and its corresponding negative control (shCtrl) were obtained from Vigene Biosciences (Vigene, Shandong, China).

### Cell culture and adipogenic differentiation

The porcine and mouse subcutaneous preadipocytes were isolated and cultured as described previously. Briefly, subcutaneous fat cells were harvested from three 3-day-old male Duroc × Landrace × Yorkshire (DLY) pigs. Inguinal white adipose tissue (iWAT) was obtained from six 1-month-old male C57BL/6 mice. Harvested fat was minced and digested in 1.5 mg/ml collagenase type I (Life Technologies Corporation, Grand Island, USA) at 37 °C for 1.5 h. The digestions were terminated with Dulbecco’s modification of Eagle’s medium (DMEM) containing 10% fetal bovine serum (FBS) (Gibco, CA, USA) and filtered through 70 μm filters to remove connective tissues and undigested trunks of tissues. Cells were washed twice with phosphate-buffered saline (PBS) by centrifugation at 450g for 5 min. The freshly isolated cells were seeded and cultured in a growth medium containing DMEM, 20% FBS, and 1% penicillin/streptomycin (Invitrogen) at 37°C with 5% CO_2_, followed by feeding with fresh medium every 2 days. The HEK293T cell line was cultured under the same conditions.

After reaching 90% confluence, subcutaneous preadipocytes were induced to differentiate with an induction medium (IM) containing DMEM, 10% FBS, 10 μg/ml insulin, 1 μM dexamethasone (DEXA), and 0.5 mM 3-isobutyl-methylxanthine (IBMX) for 3 days and then differentiated in differentiation medium (DM) contains DMEM and 10% FBS, 1 μg/ml insulin for 2 days until adipocytes mature.

### Oil red O and BODIPY staining

To quantify lipid accumulation in subcutaneous preadipocytes, Oil red O (ORO) and BODIPY staining were performed on day 5 of adipogenic differentiation. Cultured cells were washed with PBS and fixed with 4% formaldehyde for 15 min at room temperature. Then, the cells were stained using the ORO working solutions containing 6 ml ORO stock solution (5 g l-1 in isopropanol) and 4 ml ddH_2_O for 30 min. After staining, the cells were washed with 60% isopropanol in PBS and pictured. ORO dye was extracted from stained adipocytes with 100% isopropanol, and the Oil red signal was quantified by measuring the optical density at 490 nm (OD 490).

For BODIPY staining, intracellular lipids were visualized by staining with 0.5 nM BODIPY FL (Invitrogen) for 10 min. Cells were fixed afterward with 4% PFA and were examined by fluorescence microscopy.

### Quantitative real-time PCR (qPCR)

Total RNA was extracted from cells or tissues using Trizol Reagent (Invitrogen, CA, USA) and following the manufacturer’s instructions. The purity and concentration of total RNA were measured by a spectrophotometer (Nanodrop 2000; Thermo Fisher Scientific) at 260 and 280 nm. Absorption rates (260/280 nm) of all samples were between 1.8 and 2.0. Then, the first-strand cDNA was synthesized using random primers with a reverse transcription kit (Invitrogen, USA). qPCR was carried out with the CFX96 Touch Real-Time PCR detection system (Bio-Rad, Berkeley, CA, USA) using SYBR Green Master Mix (Yeasen Biotech Co., Ltd., Shanghai, China). The 2^−ΔΔCT^ method was used to analyze the relative changes in gene expression normalized against 18S rRNA as an internal control. The primer sequences used are provided in Additional file 6: Table S1.

### Protein extraction and western blotting

Total protein was isolated from cells or tissues using RIPA buffer supplemented with protease inhibitor cocktail (Thermo Fisher Scientific, Waltham, MA, USA). The protein concentration was determined with the BCA protein assay (Thermo Fisher Scientific, USA). Protein separation and western blot analysis were conducted as described earlier [5]. GADD45A antibody (sc-6850, 1: 1,000) was from Santa Cruz Biotechnology (Santa Cruz). Perilipin-1 (ab61682, 1:2000) and UCP1 (ab10983, 1:1000) were from Abcam. FABP4 (E71703-98, 1:2000) and GAPDH (EM1101, 1:5000) were from HuaBio. Cocktail (45-8099, 1:2000) was from Thermo Fisher Scientific. Stat1 (D4Y6Z, 1: 1000) and Phospho-Stat1 (Tyr701) (58D6, 1:1000) were obtained from Cell Signaling Technology. The horseradish peroxidase (HRP)-conjugated secondary antibody (anti-rabbit IgG, 111-035-003 or anti-mouse IgG; 115-035-003, Jackson ImmunoResearch) was diluted 1:10,000. Immunodetection was performed using enhanced chemiluminescence western blotting substrate (Google Biotechnology, Wuhan, Hubei, China) and detected by ChemiScope3500 Mini System.

### Co-immunoprecipitation (Co-IP) assay

Total protein was extracted from differentiated adipocytes or HEK293T cells. The lysate was precleared with protein A/G agarose at 4 °C for 1 h. Then, 2 mg of primary antibody anti-GADD45A (Santa Cruz, sc-6850) or anti-Stat1 (Cell Signaling Technology, D4Y6Z) was added into lysate containing 500 mg total protein and rotating at 4 °C overnight. The following day the protein A/G agarose was added and rotated for 2 h. The samples were washed with cold PBS three times, and protein complexes bound to the antibody were eluted and examined by western blotting.

### Luciferase assay

HEK293T cells were seeded into 24-well plates for 24 h and then transfected with different plasmids using Lipofectamine 2000 (Invitrogen, USA). The pGL3-Lkb1 promoter-luciferase plasmid was generated. For transfection of each well, Renilla plasmid (pRL-TK), pGL3-Lkb1, GADD45A or Stat1 overexpressing plasmid, and blank control plasmid were co-transfected following the manufacturer’s instructions. Cells were harvested 36 h after transfection and analyzed with the dual-luciferase reporter assay system (Promega).

### Statistical analysis

All data are presented as means ± standard errors of the means (SEM) from at least three independent experiments. In principle, after confirming a normal (Gaussian) distribution, experimental comparisons were performed using a two-tailed Student’s *t*-test, including and estimating differences between two groups. Comparisons were made by unpaired two-tailed Student’s *t* tests. Statistical significance was considered significant at *P* < 0.05. GraphPad (Prism 9) was used for data analyses.

### Supplementary Information

**Additional file 1:** **Fig. S1.** The qPCR analysis of subcutaneous fat in Shaziling and Yorkshire pigs at different growth stages. (A-C) Relative mRNA expression of marker genes for mature adipocyte (*FABP4* and *LEP*) (A), adipogenic differentiation (*CEBPα*) (B), and fatty acid synthesis (*FASN*) (C) (*n* = 6). Error bars represent SEM, * *P* < 0.05, ** *P* < 0.01, *** *P* < 0.001, two-tailed Student’s t-test.

**Additional file 2:** **Fig. S2.** GADD45A influences adipogenic differentiation and lipid accumulation in porcine subcutaneous adipocytes. (A) ORO and BODIPY staining of total lipids in undifferentiated and differentiated porcine subcutaneous adipocytes. OD490 was measured (*n* = 4). (B) The expression of *GADD45A* and *FABP4* (*n* = 5), *PPARγ*, *ADIPOQ*, and *LEP* (*n* = 3) was assessed by qPCR. (C) Western blotting analysis. (D) Porcine subcutaneous adipocytes were infected with control adenovirus (Ctrl) and adenovirus-expressing GADD45A (G45a-oe) and were allowed to differentiate. ORO and BODIPY staining of control and G45a-oe cells five days after induction of differentiation. OD490 was measured based on ORO (*n* = 4). (E) Relative mRNA levels of GADD45A and adipogenic-related genes in control and G45a-oe cells after differentiation (*n* = 3 or *n* = 4). (F) Protein levels of Perilipin-1 and FABP4 with GADD45A OE. (G) ORO staining of total lipids in differentiated porcine subcutaneous adipocytes infected with adenovirus control shRNA (shCtrl) and adenovirus GADD45A shRNA (shG45a) (*n* = 6). (H, I) The mRNA and protein levels of related genes with GADD45A KD after differentiation (*n* = 3). Error bars represent SEM, *P<0.05, **P<0.01, ***P<0.001, two-tailed Student’s t-test. Scale bars: 200 μm and 100 μm.

**Additional file 3:** **Fig. S3.** (A-D) Quantification of adipocyte area in Fig. 4M (A, B) and Fig. 4N (C, D) using Image J software (*n* = 3). Error bars represent SEM, *P<0.05, **P<0.01,***P<0.001, two-tailed Student’s t-test.

**Additional file 4:** **Fig. S4.** GADD45A deletion promotes Lkb1 expression. (A, B) Protein (A) and mRNA (B) levels of Lkb1 in iWAT of *Gadd45a*^*-/-*^ mice (*n* = 4). Error bars represent SEM, *P<0.05,**P<0.01, ***P<0.001, two-tailed Student’s t-test.

**Additional file 5:** **Fig. S5.** Reanalysis of GADD45A expression in different types of human adipocytes from published data. (A) Log2 fold changes of thermogenic genes and Gadd45a in human BAT and WAT (GSE113764). (B) Log2 fold changes of related genes from human adipocytes derived from non-thermogenic and thermogenic adipose tissue deposits, namely abdominal subcutaneous adipose tissue (AbdSQ) and supraclavicular adipose tissue (SClav) (GSE150119). (C) Log2 fold changes of genes in human adipocytes with (FSK) or without (Vehicle) Forskolin treatment (GSE65190).

**Additional file 6: Table. S1.** The primers sequence.

**Additional file 7. **Full scans of immunoblots.

**Additional file 8. **Individual data values.

## Data Availability

All datasets generated or analyzed during this study are included in this published article and its supplementary information files. The individual data values for Figs 2, 3, 5, and 6, as well as Additional file 2: Fig. S2, Additional file 3: Fig. S3, Additional file 4: Fig. S4, and Additional file 5: Fig. S5 are provided in Additional file 8.

## Abbreviations

AMPK: Adenosine monophosphate-activated protein kinase
BAT: Brown adipose tissue
Co-IP: Co-immunoprecipitation
DEGs: Differentially expressed genes
DEXA: Dexamethasone
DLY: Duroc × Landrace × Yorkshire
DM: Differentiation medium
DMEM: Dulbecco’s modification of Eagle’s medium
FBS: Fetal bovine serum
GADD45A: Growth arrest and DNA damage-inducible A
GAS: Gastrocnemius muscle
GTT: Glucose tolerance tests
H&E: Hematoxylin and eosin
HFD: High-fat diet
IBMX: 3-Isobutyl-methylxanthine
IM: Induction medium
ITT: Insulin tolerance tests
iWAT: Inguinal white adipose tissue
KO: Knockout
LDs: Lipid droplets
Lkb1: Liver kinase b1
MS: Mass spectrometry
ORO: Oil red O
PBS: Phosphate-buffered saline
PFA: Paraformaldehyde
SAT: Subcutaneous adipose tissue
Stat1: Signal transducer and activator of transcription 1
STK11: Serine/threonine kinase 11
TA: Tibialis anterior muscle
TC: Total cholesterol
TGs: Triglycerides
UCP1: Uncoupling protein 1
WAT: White adipose tissue
WCL: Whole cell lysate
WT: Wild-type
