# Supplementary figures and images for: GADD45A regulates subcutaneous fat deposition and lipid metabolism by interacting with Stat1
Source: BMC Biol. 2023 Oct 9;21:212. doi: 10.1186/s12915-023-01713-z (PMC10561432; doi:10.1186/s12915-023-01713-z)

Fig. S1

A

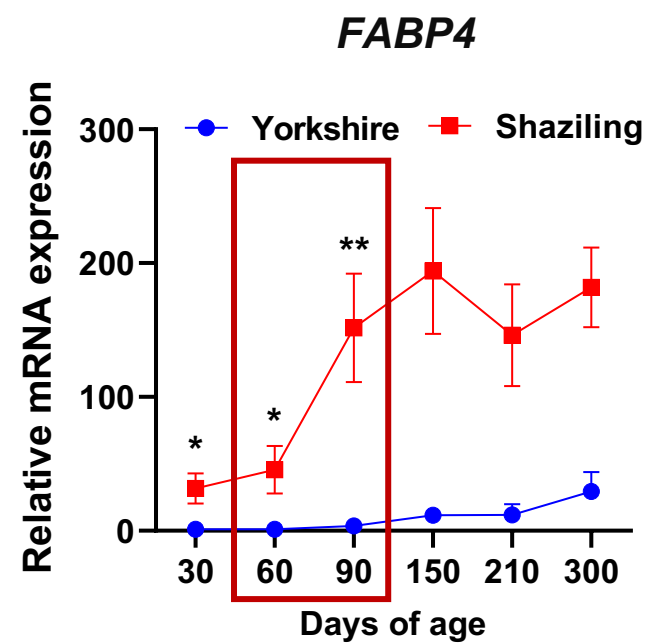

*LEP*

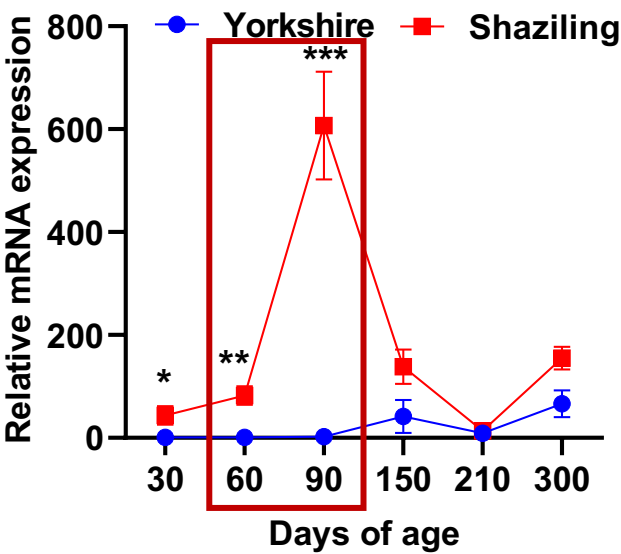

B

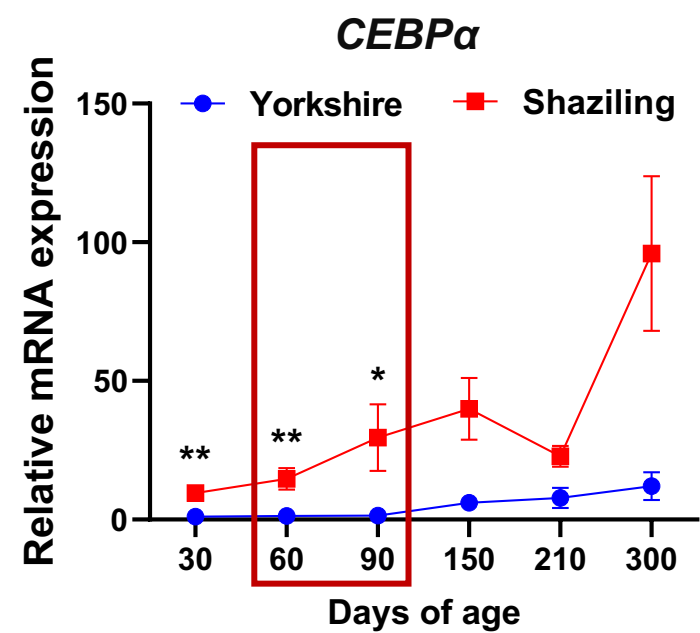

C

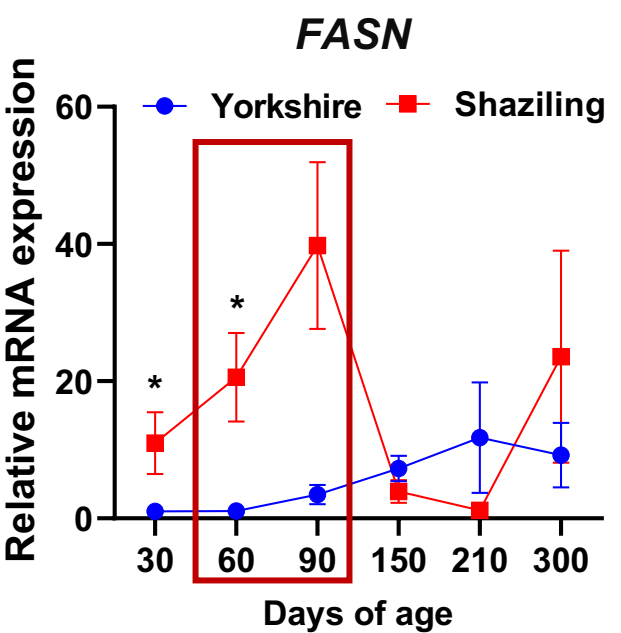

Supplement: Supplementary file 1 — Additional file 1: Fig. S1. The qPCR analysis of subcutaneous fat in Shaziling and Yorkshire pigs at different growth stages. (A-C) Relative mRNA expression of marker genes for mature adipocyte (FABP4 and LEP) (A), adipogenic differentiation (CEBPα) (B), and fatty acid synthesis (FASN) (C) (n = 6). Error bars represent SEM, * P < 0.05, ** P < 0.01, *** P < 0.001, two-tailed Student’s t-test. [file 12915_2023_1713_MOESM1_ESM.pdf]

Fig. S3

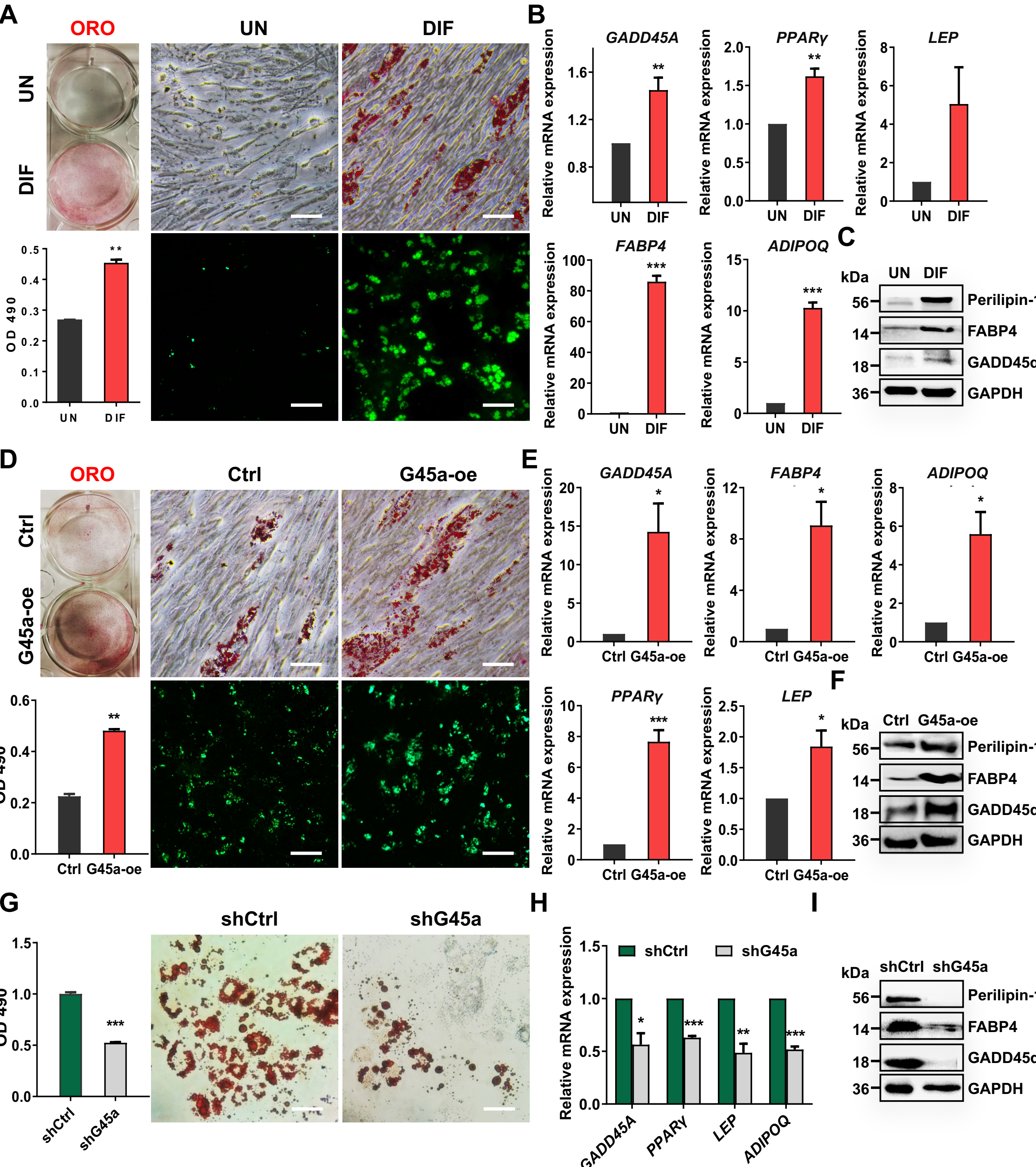

Supplement: Supplementary file 2 — Additional file 2: Fig. S2. GADD45A influences adipogenic differentiation and lipid accumulation in porcine subcutaneous adipocytes. (A) ORO and BODIPY staining of total lipids in undifferentiated and differentiated porcine subcutaneous adipocytes. OD490 was measured (n = 4). (B) The expression of GADD45A and FABP4 (n = 5), PPARγ, ADIPOQ, and LEP (n = 3) was assessed by qPCR. (C) Western blotting analysis. (D) Porcine subcutaneous adipocytes were infected with control adenovirus (Ctrl) and adenovirus-expressing GADD45A (G45a-oe) and were allowed to differentiate. ORO and BODIPY staining of control and G45a-oe cells five days after induction of differentiation. OD490 was measured based on ORO (n = 4). (E) Relative mRNA levels of GADD45A and adipogenic-related genes in control and G45a-oe cells after differentiation (n = 3 or n = 4). (F) Protein levels of Perilipin-1 and FABP4 with GADD45A OE. (G) ORO staining of total lipids in differentiated porcine subcutaneous adipocytes infected with adenovirus control shRNA (shCtrl) and adenovirus GADD45A shRNA (shG45a) (n = 6). (H, I) The mRNA and protein levels of related genes with GADD45A KD after differentiation (n = 3). Error bars represent SEM, *P<0.05, **P<0.01, ***P<0.001, two-tailed Student’s t-test. Scale bars: 200 μm and 100 μm. [file 12915_2023_1713_MOESM2_ESM.pdf]

Fig. S4

A

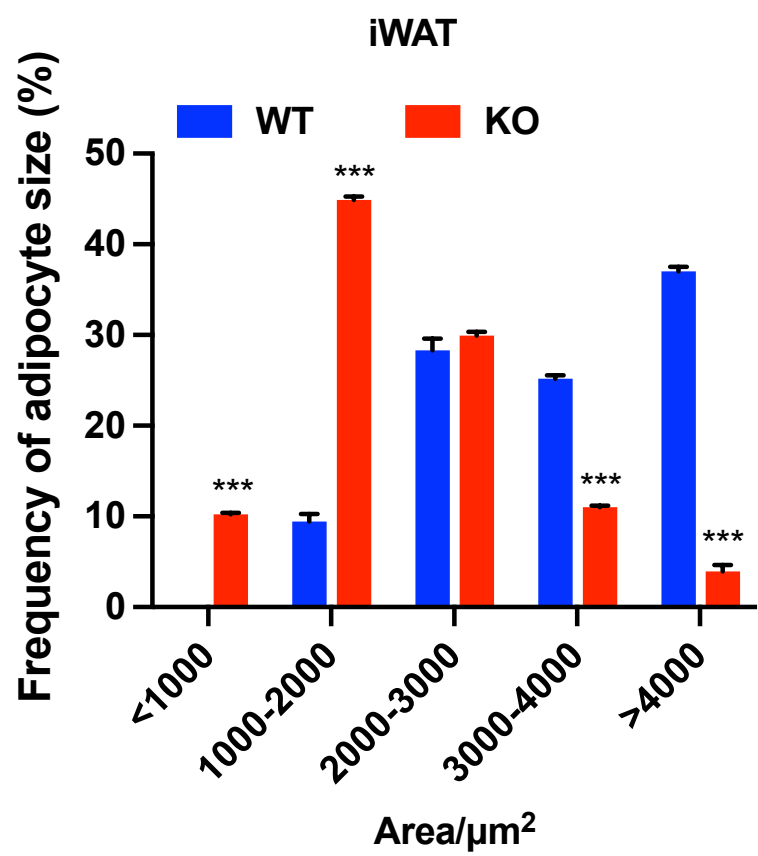

B

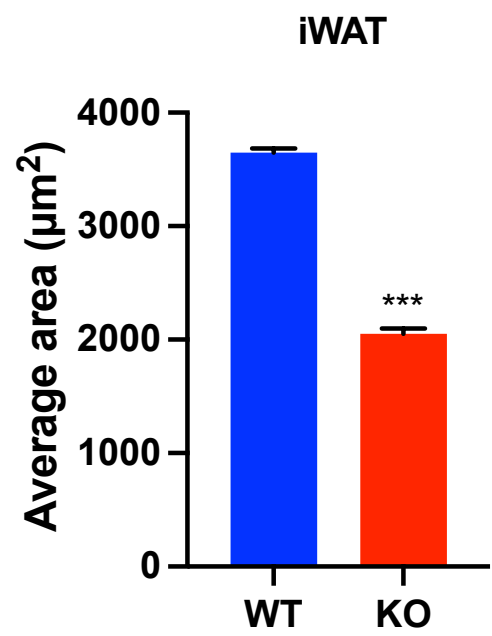

C

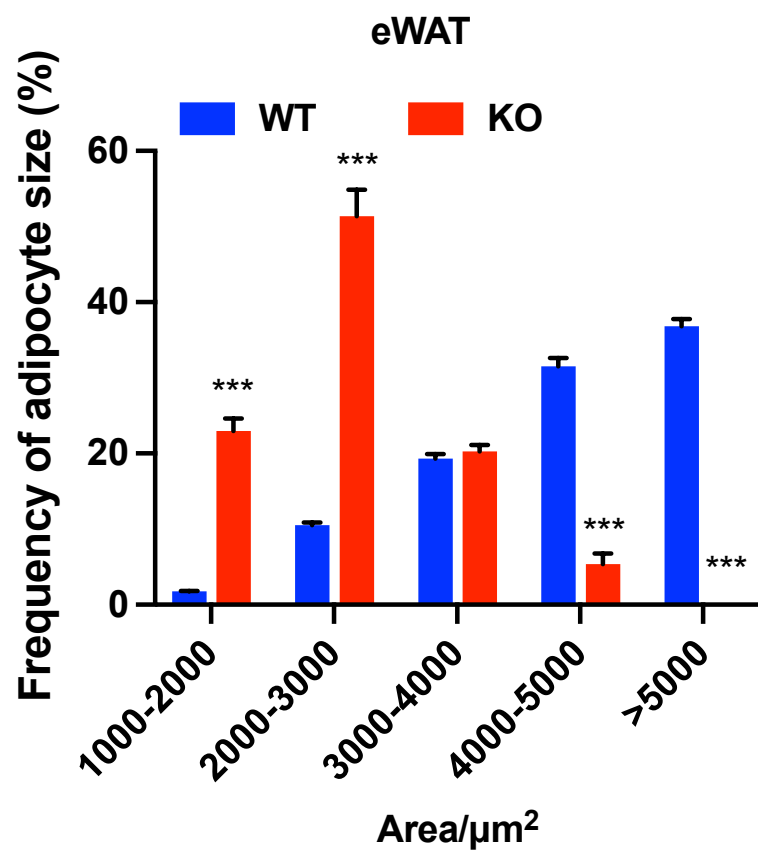

D

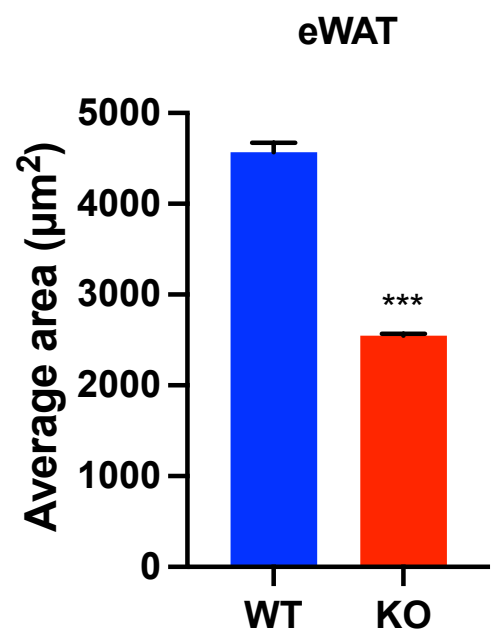

Supplement: Supplementary file 3 — Additional file 3: Fig. S3. (A-D) Quantification of adipocyte area in Fig. 4M (A, B) and Fig. 4N (C, D) using Image J software (n = 3). Error bars represent SEM, *P<0.05, **P<0.01,***P<0.001, two-tailed Student’s t-test. [file 12915_2023_1713_MOESM3_ESM.pdf]

Fig. S5

A

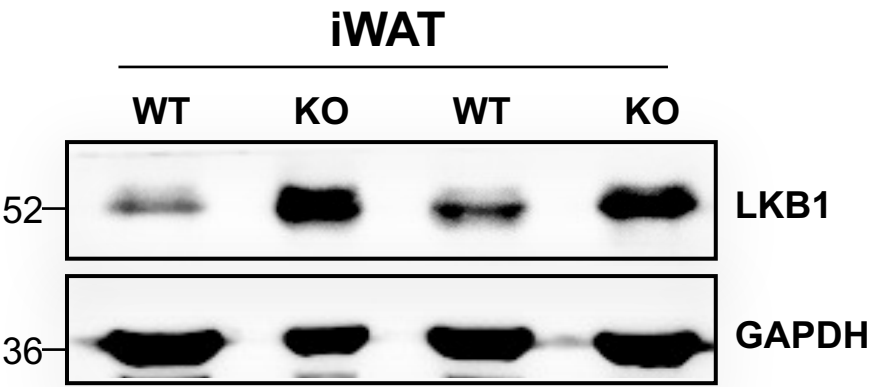

B

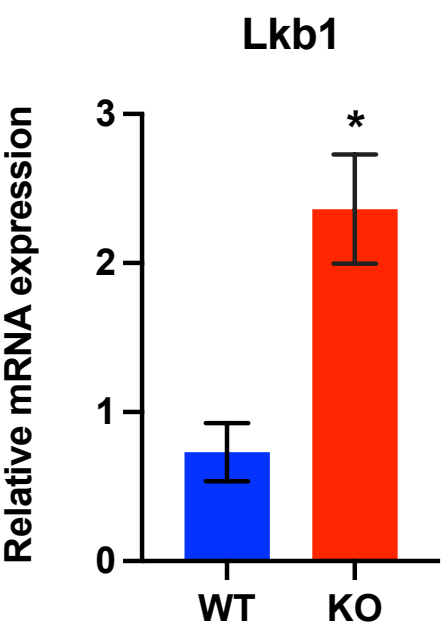

Supplement: Supplementary file 4 — Additional file 4: Fig. S4. GADD45A deletion promotes Lkb1 expression. (A, B) Protein (A) and mRNA (B) levels of Lkb1 in iWAT of Gadd45a-/- mice (n = 4). Error bars represent SEM, *P<0.05,**P<0.01, ***P<0.001, two-tailed Student’s t-test. [file 12915_2023_1713_MOESM4_ESM.pdf]

Fig. S2

A

GSE113764 BAT vs WAT (Human)

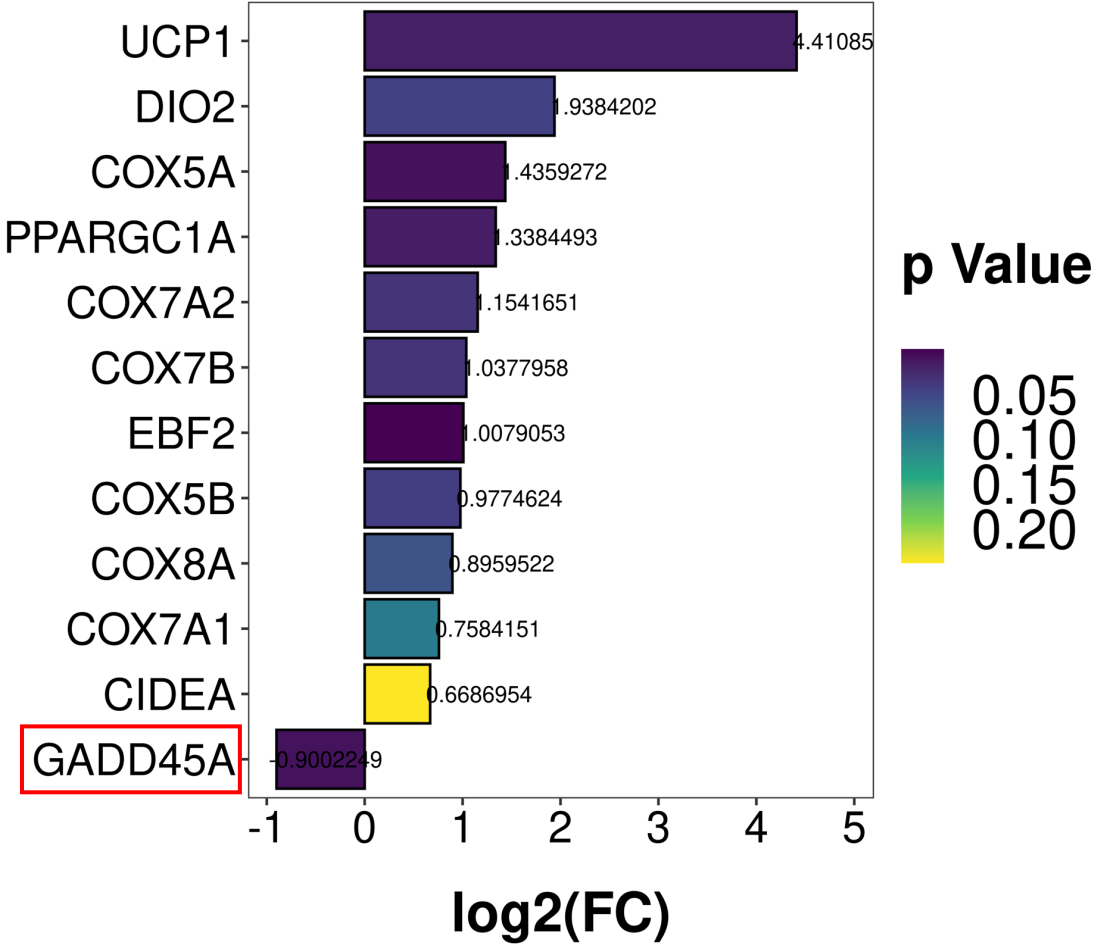

B

GSE150119 SClav vs AbdSQ (Human)

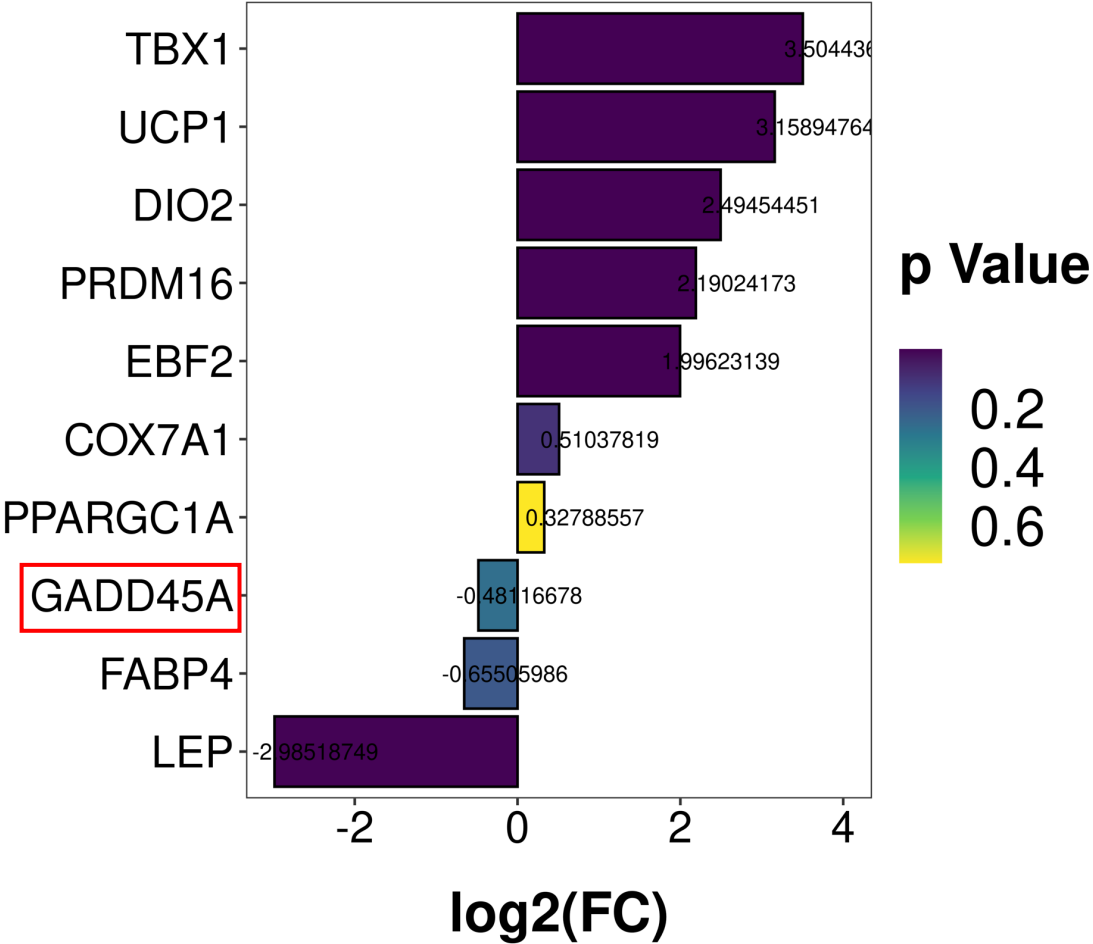

C

GSE65190 FSK vs Vehicle (Human)

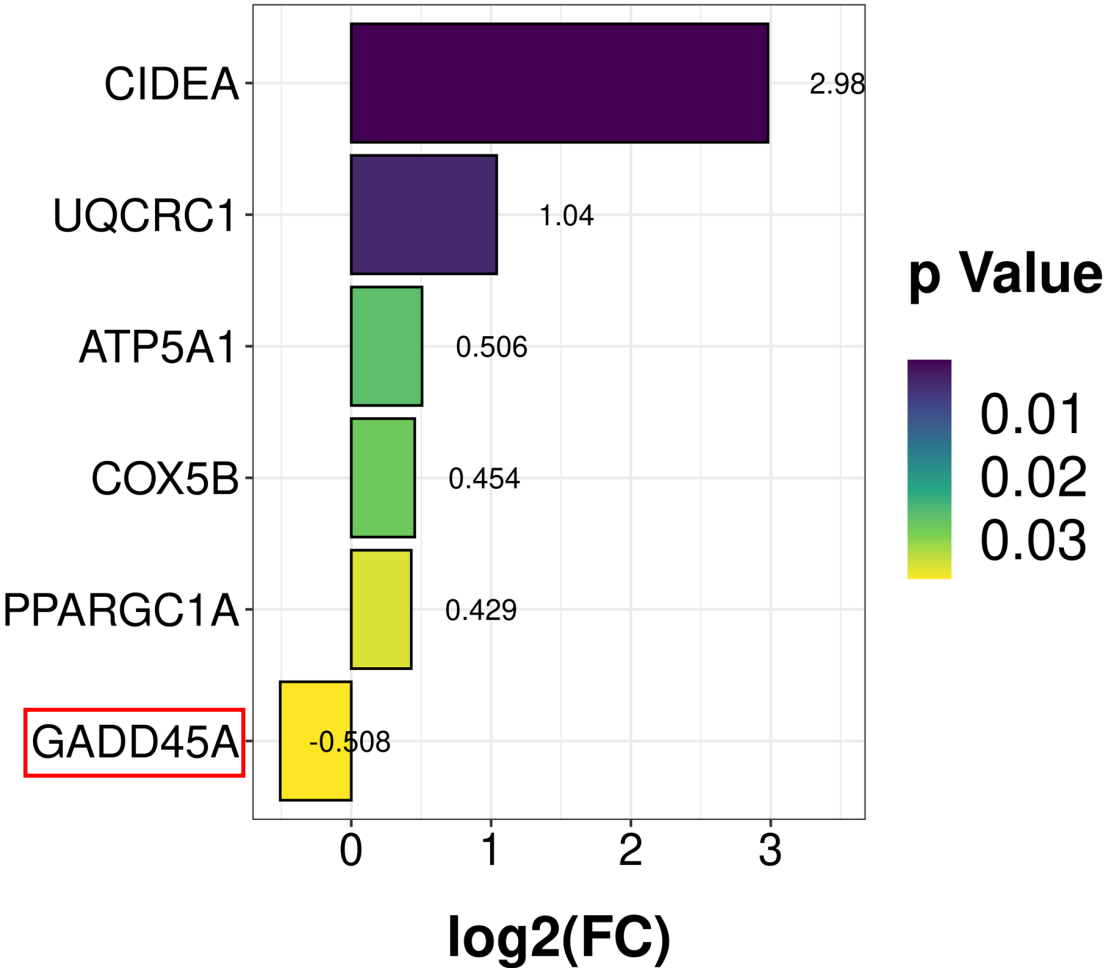

Supplement: Supplementary file 5 — Additional file 5: Fig. S5. Reanalysis of GADD45A expression in different types of human adipocytes from published data. (A) Log2 fold changes of thermogenic genes and Gadd45a in human BAT and WAT (GSE113764). (B) Log2 fold changes of related genes from human adipocytes derived from non-thermogenic and thermogenic adipose tissue deposits, namely abdominal subcutaneous adipose tissue (AbdSQ) and supraclavicular adipose tissue (SClav) (GSE150119). (C) Log2 fold changes of genes in human adipocytes with (FSK) or without (Vehicle) Forskolin treatment (GSE65190). [file 12915_2023_1713_MOESM5_ESM.pdf]

**Fig. 2G**

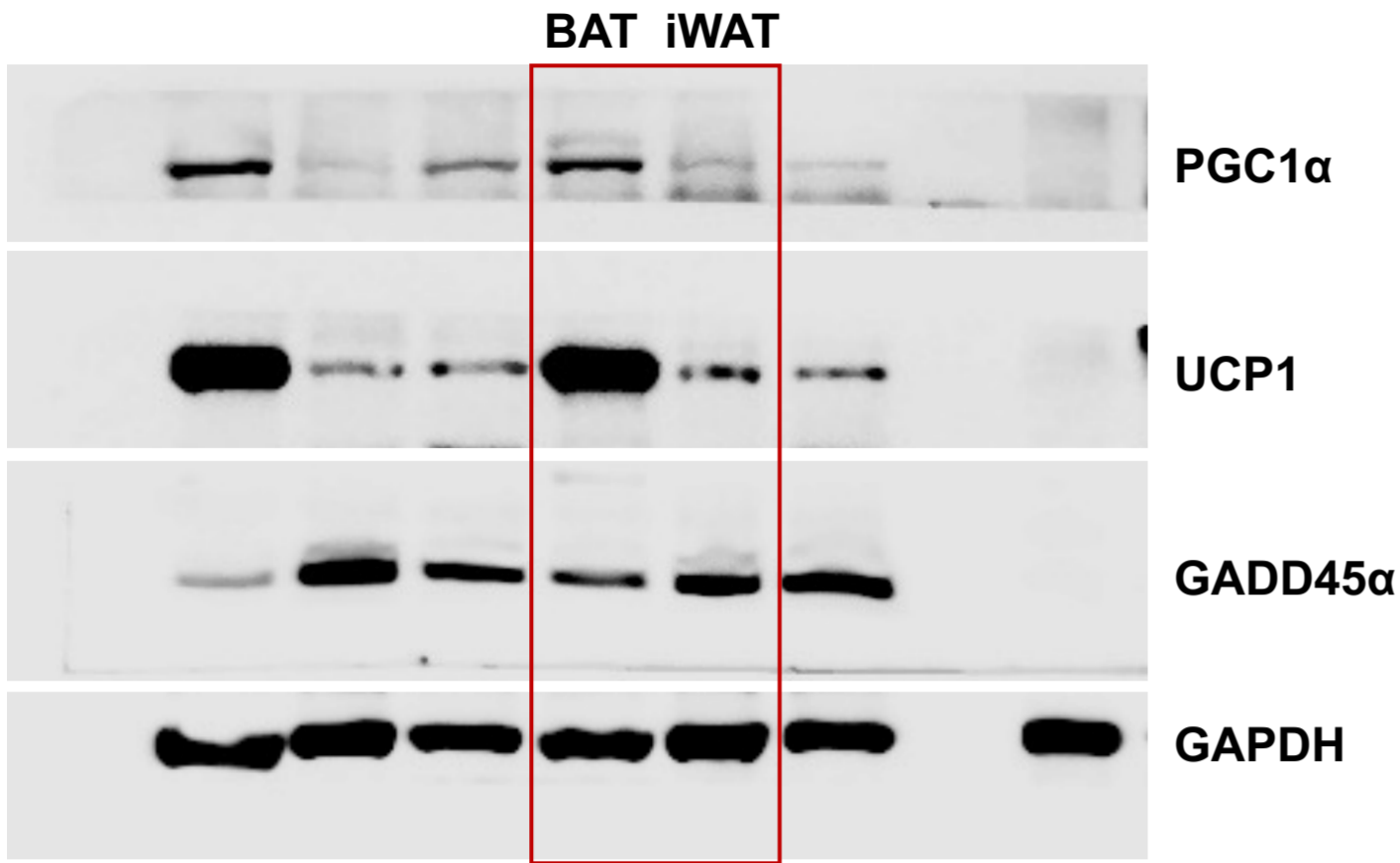

**Fig. 4C**

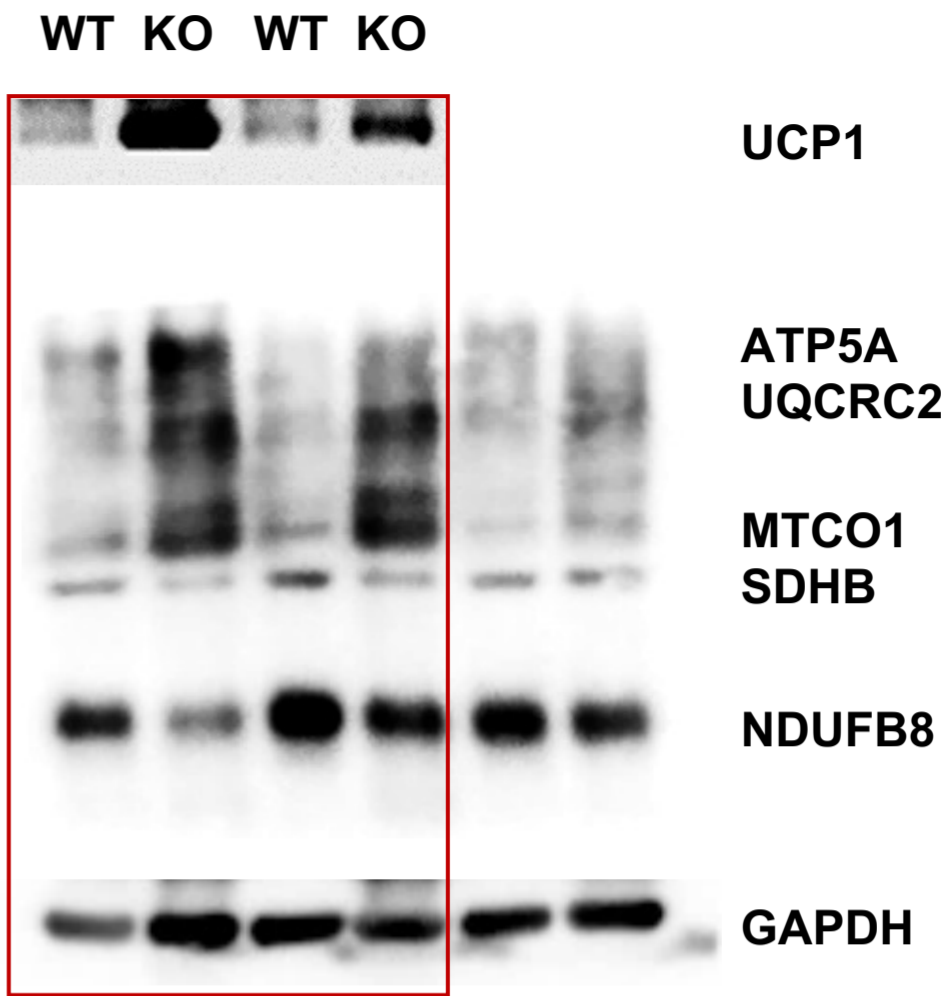

**Fig. 3C**

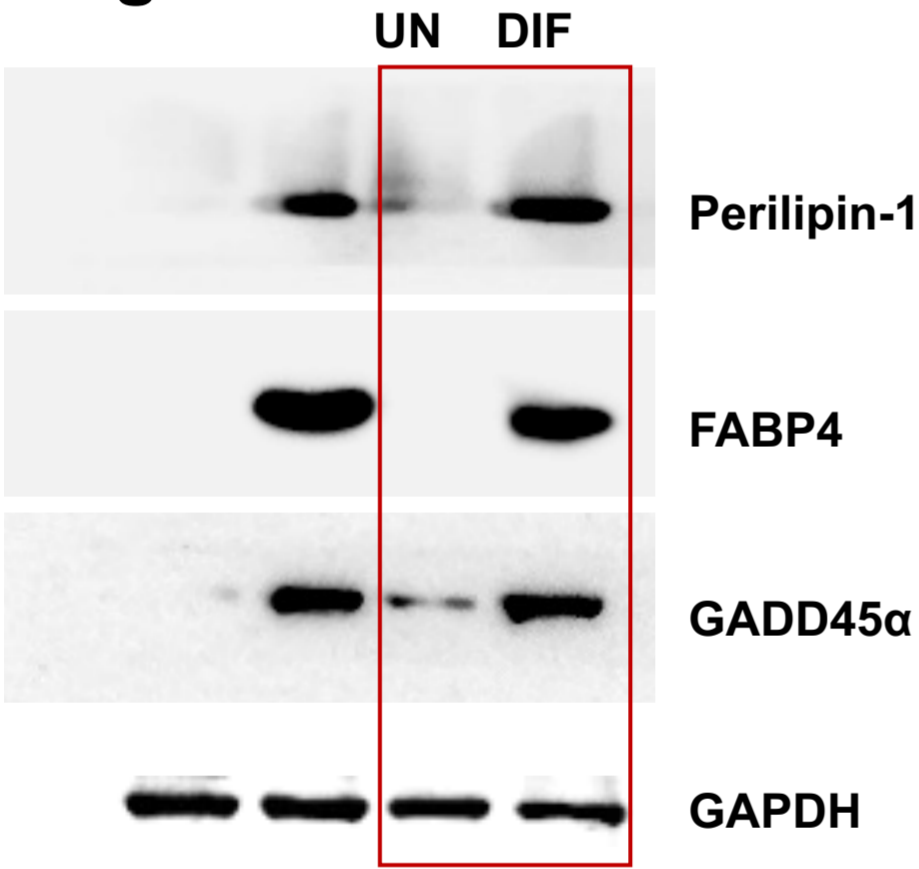

**Fig. 5C**

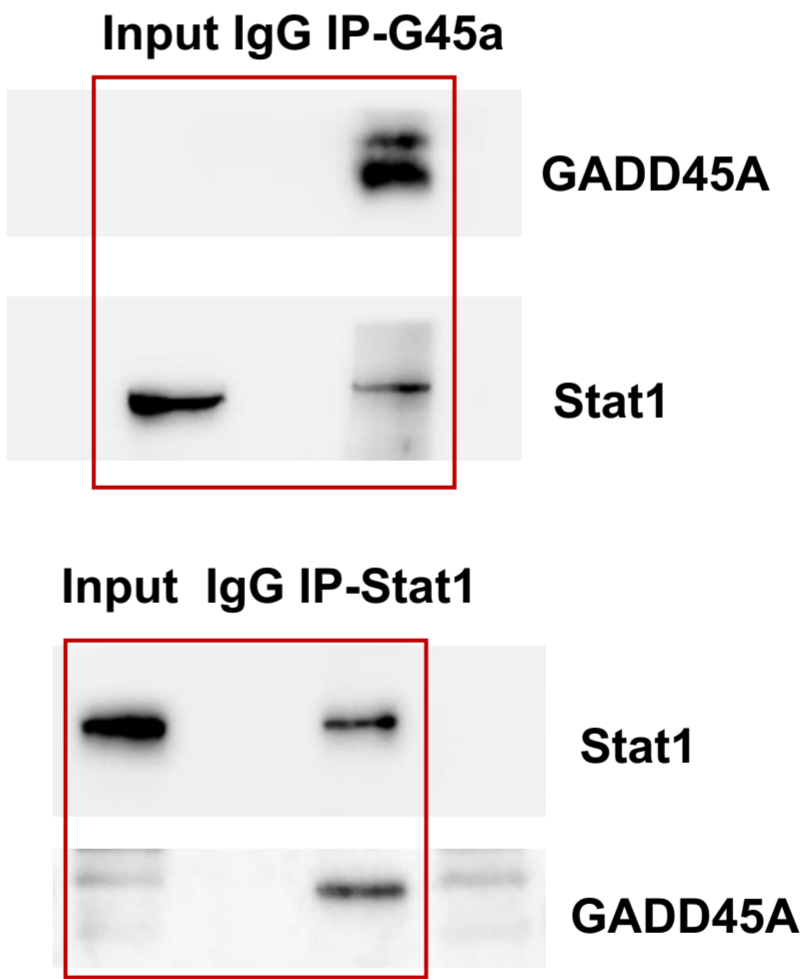

**Fig. 3F**

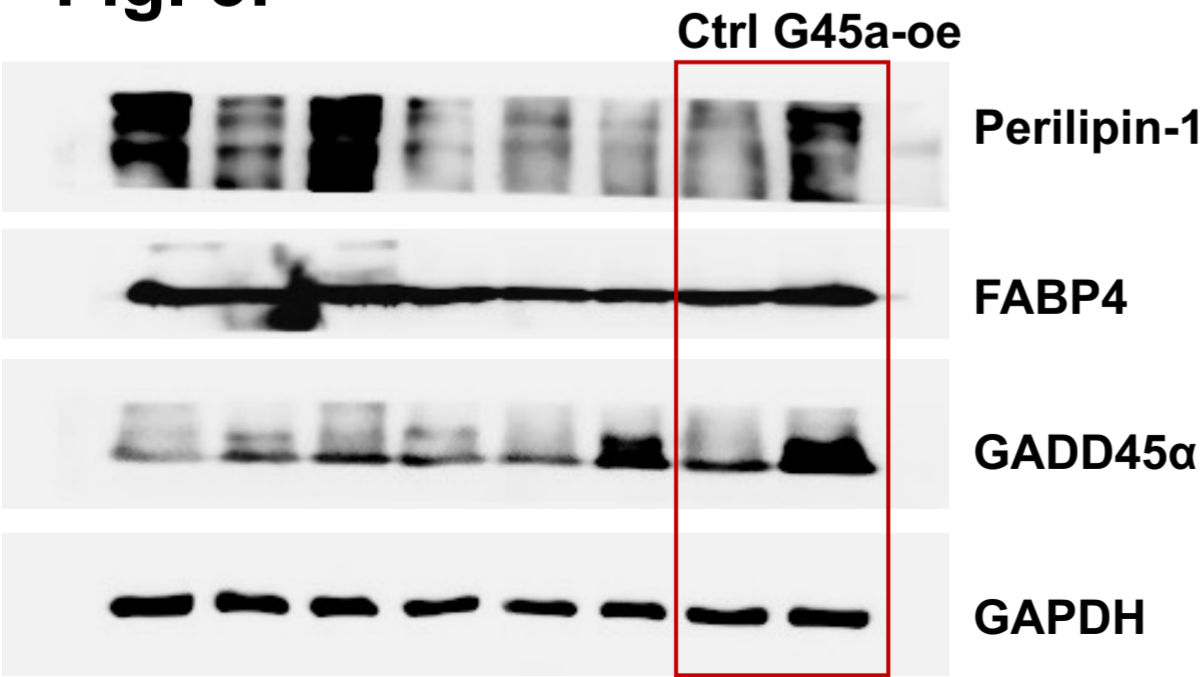

**Fig. 5D**

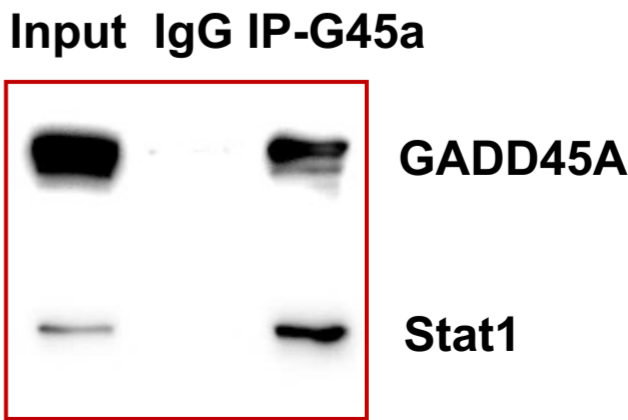

Fig. 6B

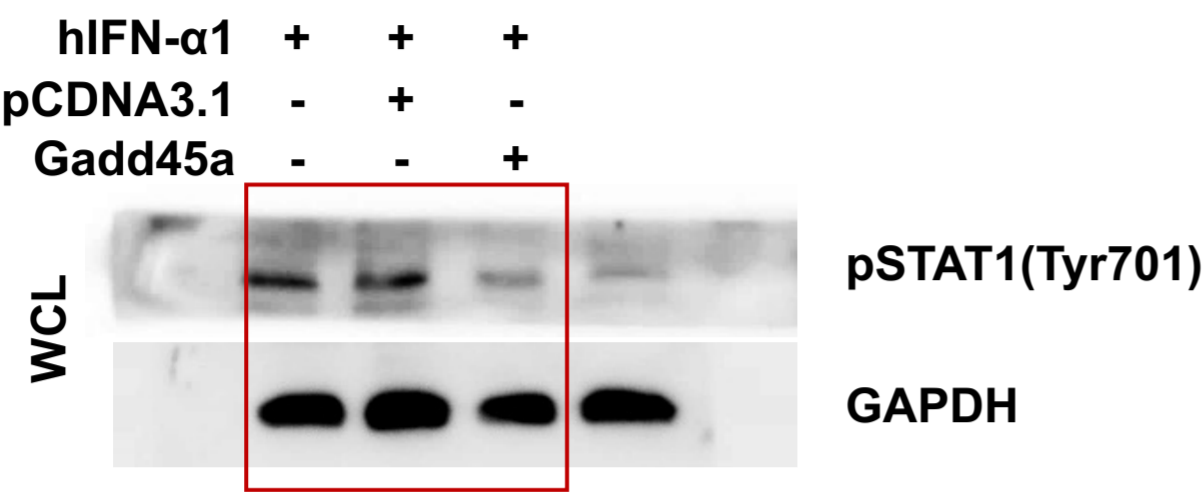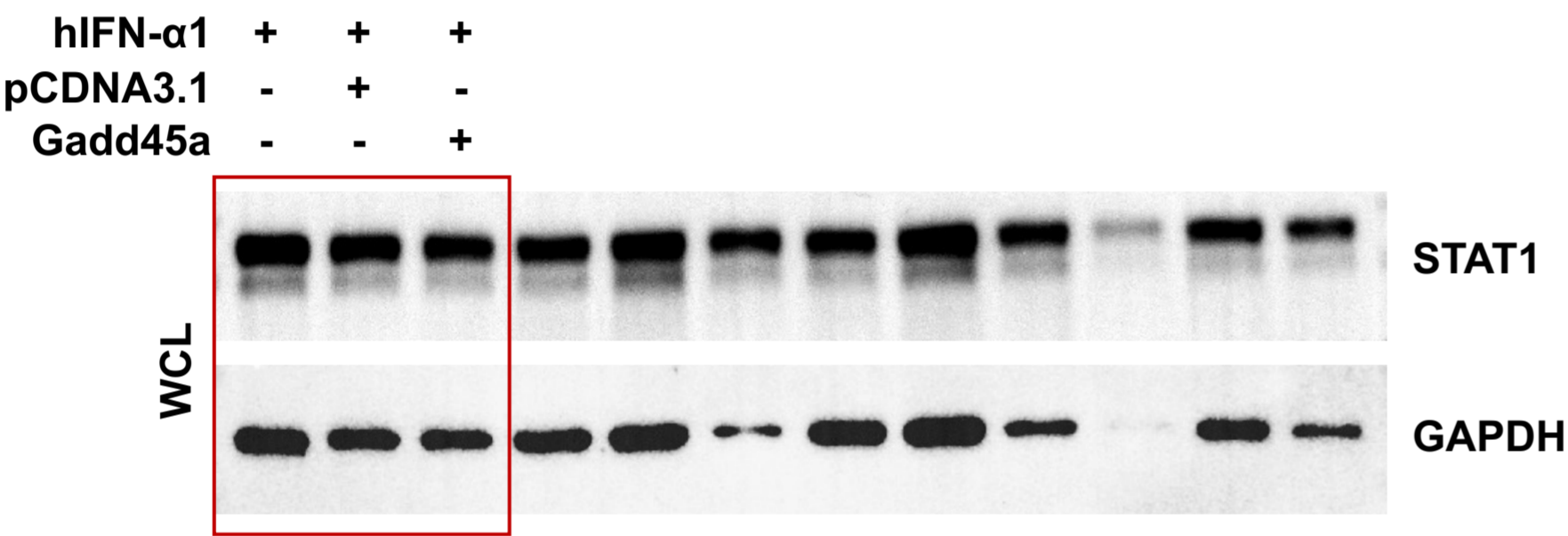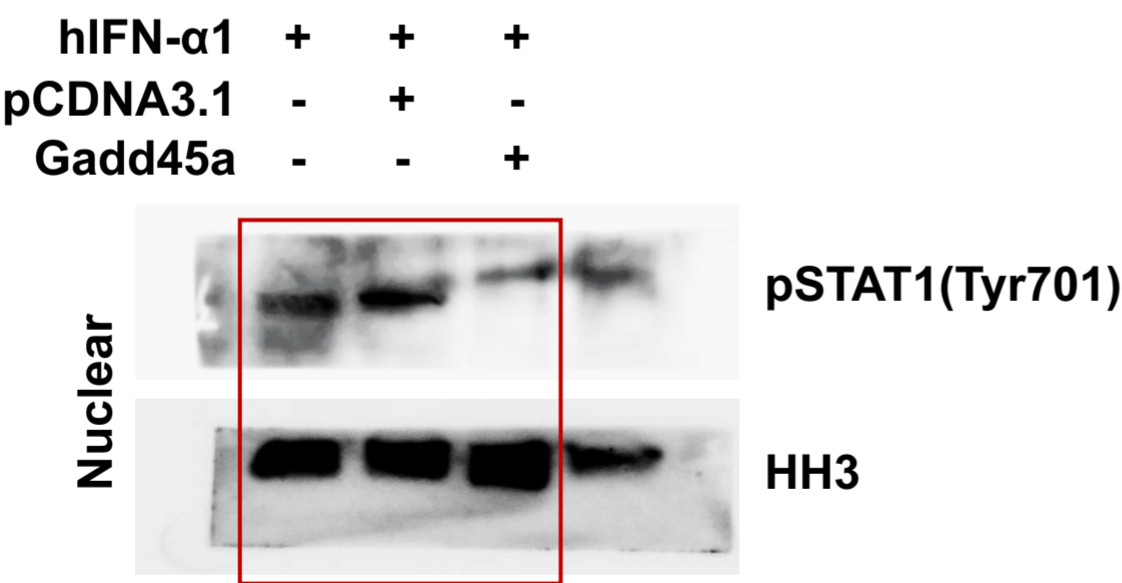

Fig. S2C

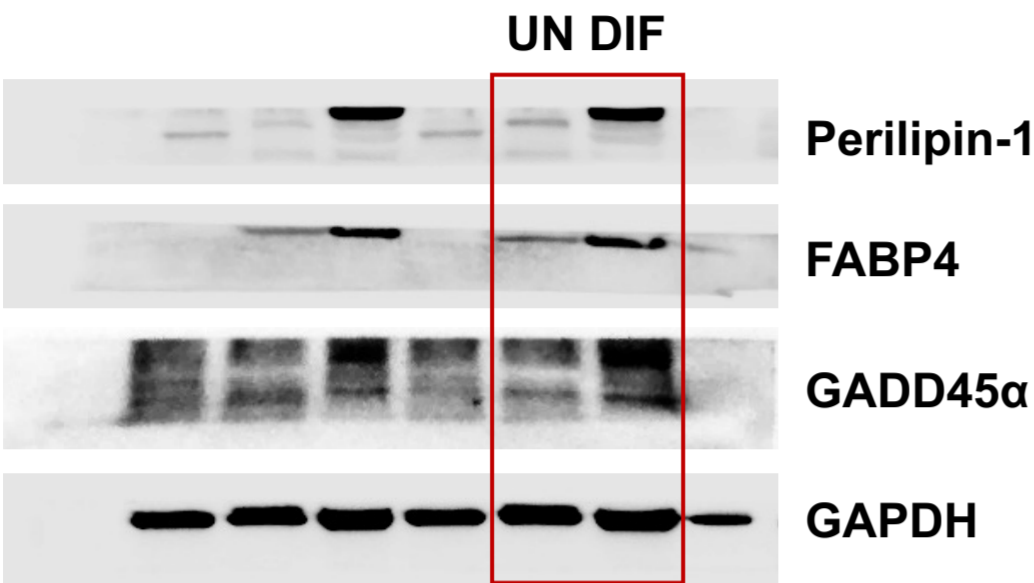

Fig. S2F

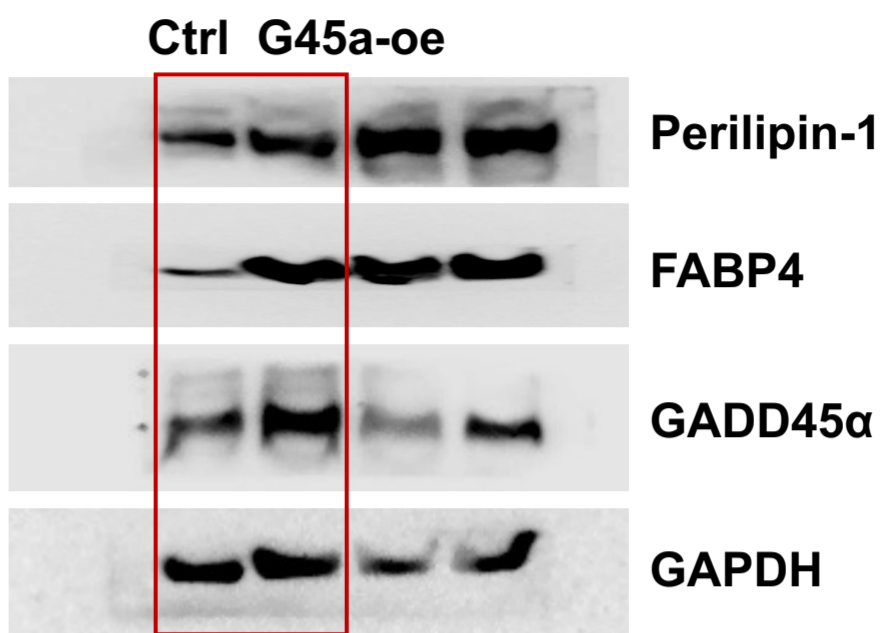

Fig. S2I

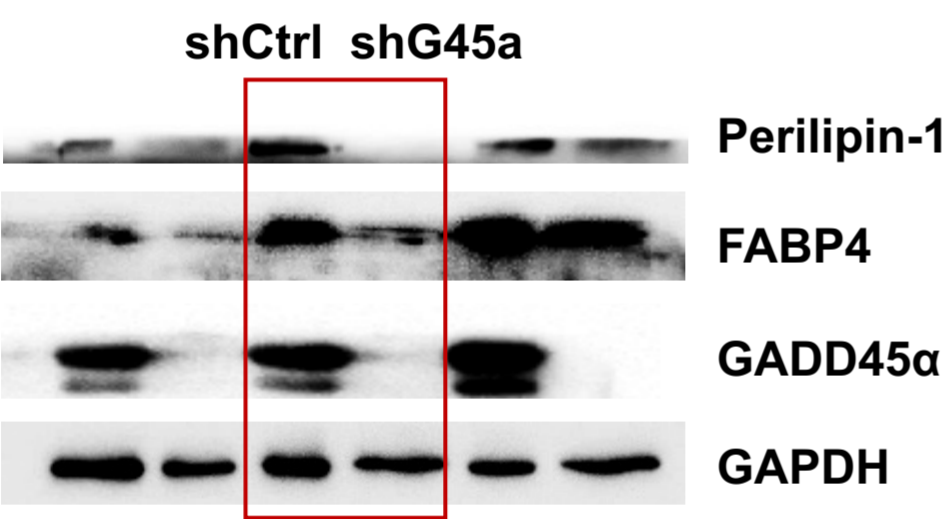

Fig. S4A

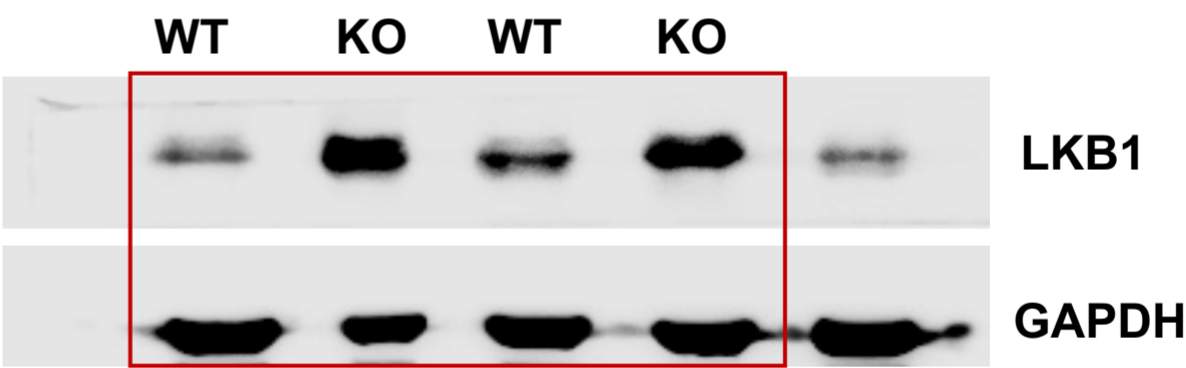

Supplement: Supplementary file 7 — Additional file 7. Full scans of immunoblots. [file 12915_2023_1713_MOESM7_ESM.pdf]
